# Topological unification of time-reversal and particle-hole symmetries in non-Hermitian physics

**DOI:** 10.1038/s41467-018-08254-y

**Published:** 2019-01-17

**Authors:** Kohei Kawabata, Sho Higashikawa, Zongping Gong, Yuto Ashida, Masahito Ueda

**Affiliations:** 1 Department of Physics, University of Tokyo, 7-3-1 Hongo, Bunkyo-ku Tokyo, 113-0033 Japan; 2 RIKEN Center for Emergent Matter Science (CEMS), Wako Saitama, 351-0198 Japan

## Abstract

Topological phases are enriched in non-equilibrium open systems effectively described by non-Hermitian Hamiltonians. While several properties unique to non-Hermitian topological systems were uncovered, the fundamental role of symmetry in non-Hermitian physics has yet to be fully understood, and it has remained unclear how symmetry protects non-Hermitian topological phases. Here we show that two fundamental anti-unitary symmetries, time-reversal and particle-hole symmetries, are topologically equivalent in the complex energy plane and hence unified in non-Hermitian physics. A striking consequence of this symmetry unification is the emergence of unique non-equilibrium topological phases that have no counterparts in Hermitian systems. We illustrate this by presenting a non-Hermitian counterpart of the Majorana chain in an insulator with time-reversal symmetry and that of the quantum spin Hall insulator in a superconductor with particle-hole symmetry. Our work establishes a fundamental symmetry principle in non-Hermitian physics and paves the way towards a unified framework for non-equilibrium topological phases.

## Abstract (web summary)

Topological phases of matter are determined by its symmetries and dimension. Here the authors show that in non-Hermitian systems, such as those with gain and loss, time-reversal and particle-hole symmetries are equivalent to each other, unifying otherwise distinct topological classes and leading to emergent non-Hermitian topological phases.

## Introduction

It was Wigner who showed that all symmetries are either unitary or anti-unitary and identified the fundamental role of time-reversal symmetry in anti-unitary operations^1^. Time-reversal symmetry is complemented by particle-hole and chiral symmetries, culminating in the Altland–Zirnbauer (AZ) ten-fold classification^2^. The AZ classification plays a key role in characterizing the topological phases^3–5^ of condensed matter such as insulators^6–12^ and superconductors^13–16^, as well as photonic systems^17^ and ultracold atoms^18^, all of which are classified into the periodic table^19–22^. Whereas the topological phase in the quantum Hall insulator is free from any symmetry constraint and breaks down in the presence of time-reversal symmetry^6,7^, certain topological phases are protected by symmetry; for example, the quantum spin Hall insulator is protected by time-reversal symmetry^8–11^ and the Majorana chain is protected by particle-hole symmetry^14^.

Despite its enormous success, the existing framework for topological phases mainly concerns equilibrium closed systems. Meanwhile, there has been growing interest in non-equilibrium open topological systems, especially non-Hermitian topological systems^23–42^. In general, non-Hermiticity arises from the presence of energy or particle exchanges with an environment^43,44^, and a number of phenomena and functionalities unique to non-conservative systems have been theoretically predicted^45–56^ and experimentally observed^57–67^. Here symmetry again plays a key role; for example, spectra of non-Hermitian Hamiltonians can be entirely real in the presence of parity-time symmetry^46^. Recently, a topological band theory for non-Hermitian Hamiltonians was developed, and the topological phase in the quantum Hall insulator was shown to persist even in the presence of non-Hermiticity^31^. Moreover, topological lasers were proposed and realized on the basis of the interplay between non-Hermiticity and topology^39,41,42^. However, it is yet to be understood how symmetry constrains non-Hermitian systems in general and how symmetry protects non-Hermitian topological phases.

Here we point out that two fundamental anti-unitary symmetries, time-reversal symmetry and particle-hole symmetry, are the two sides of the same symmetry in non-Hermitian physics. In fact, once we lift the Hermiticity constraint on the Hamiltonian *H*, the Wigner theorem dictates that an anti-unitary operator $\mathcal{A}$ is only required to satisfy

1 $${\mathcal{A}} H {\mathcal{A}}^{ - 1} = e^{{\mathrm{i}}\varphi }H\quad\left( {0 \le \varphi \, < \, 2\pi } \right).$$

This suggests that time-reversal symmetry (*φ* = 0) and particle-hole symmetry (*φ* = *π*) can be continuously transformed into each other in the complex energy plane. This topological unification leads to striking predictions about topological phenomena. In particular, properties intrinsic to topological insulators can appear also in the corresponding topological superconductors, and vice versa: a counterpart of the Majorana chain in a non-Hermitian insulator with time-reversal symmetry and that of the quantum spin Hall insulator in a non-Hermitian superconductor with particle-hole symmetry. We emphasize that such topological phases are absent in Hermitian systems; non-Hermiticity alters the topological classification in a fundamental manner, and non-equilibrium topological phases unique to non-Hermitian systems emerge as a result of the topological unification of time-reversal and particle-hole symmetries.

## Results

### Symmetries and complex spectra

**Fig. 1:**
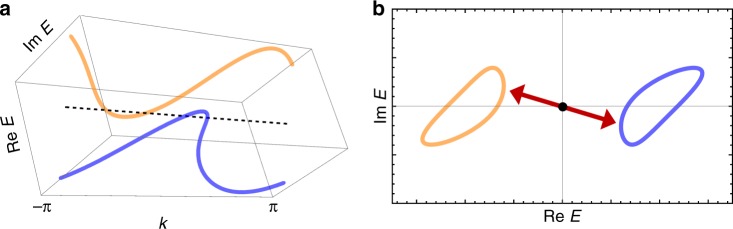
Gapped band structure for a non-Hermitian Hamiltonian. Energy dispersion for two bands (blue and orange curves) in one dimension: **a** (*k*, Re *E*, Im *E*) with wavenumber *k* and complex energy *E* and **b** its projection on the complex energy plane (Re *E*, Im *E*). The two bands neither touch nor intersect for any *k*, and therefore they are gapped. This definition does not distinguish between real and imaginary parts of energy

To go beyond the Hermitian paradigm, it is necessary to revisit some fundamental concepts relevant to topology. We start by defining a gapped complex band. Let us consider a complex-band structure $\{ E_n({\boldsymbol{k}}) \in {\Bbb C}\}$, where ***k*** is a crystal wavevector in the Brillouin zone and *n* is a band index. Since a band gap should refer to an energy range in which no states exist, it is reasonable to define a band *n* to be gapped such that *E*_*m*_ (***k***) ≠ *E*_*n*_ (***k***) for all the band indices *m* ≠ *n* and wavevectors ***k*** (Fig. 1)^31^, which is a natural generalization of the gapped band structure in the Hermitian band theory and explains the experimentally observed topological edge states in non-Hermitian systems^36–39,41,42^. Notably, the presence of a complex gap has a significant influence on the non-equilibrium wave dynamics (see Supplementary Note 1 for details). This definition of a complex gap is distinct from that adopted in ref. ^68^ and hence the corresponding topological classification is different.

**Fig. 2:**
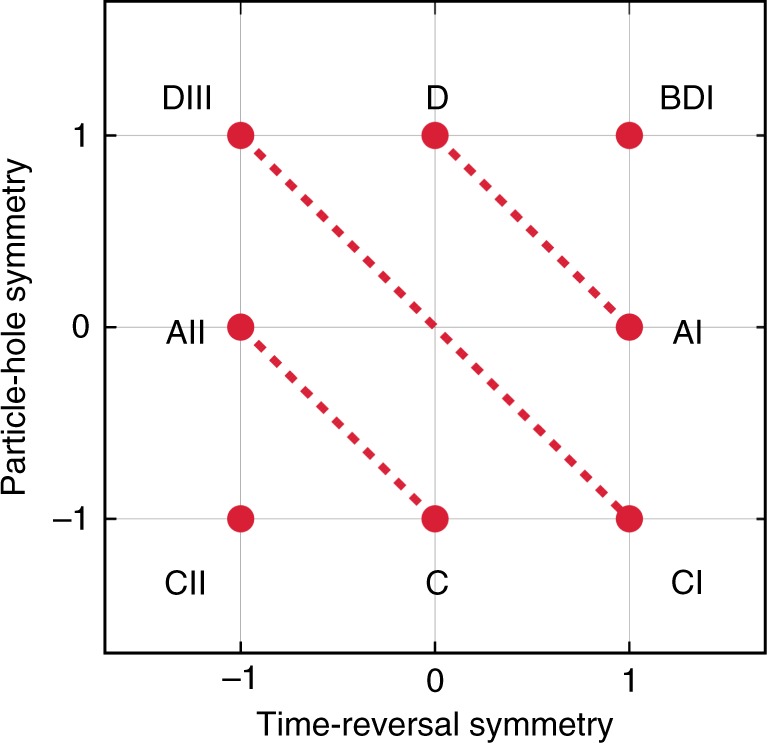
Altland–Zirnbauer symmetry classes that involve the anti-unitary symmetry (real class). The classes are specified by the values of ${\cal T}^2 = \pm 1$ and ${\cal C}^2 = \pm 1$, and the absence of symmetry is indicated by 0. The class symbol follows Cartan’s notation. While there are eight symmetry classes in Hermitian physics, the classes connected by dotted lines are topologically equivalent and thus the symmetry classes reduce to five in non-Hermitian physics. For instance, a non-Hermitian Hamiltonian having time-reversal symmetry with ${\cal T}^2 = + 1$ alone (class AI) and that having particle-hole symmetry with ${\cal C}^2 = + 1$ alone (class D) are equivalent and hence unified

We next consider the constraints on complex spectra imposed by anti-unitary symmetry (Table 1). A Hamiltonian *H* has time-reversal and particle-hole symmetries if and only if there exist anti-unitary operators $\mathcal{T}$ and $\mathcal{C}$ such that

2 $${\cal T}H{\cal T}^{ - 1} = H,\quad {\cal C}H{\cal C}^{ - 1} = - H,$$

and ${\cal T}\,z\,{\cal T}^{ - 1} = z^ \ast$, ${\cal C}\,z\,{\cal C}^{ - 1} = z^ \ast$ for all $z \in {\Bbb C}$. For Hermitian Hamiltonians with entirely real spectra, time-reversal symmetry places no constraints on the real spectra and particle-hole symmetry renders the real spectra symmetric about zero energy. By contrast, for non-Hermitian Hamiltonians, of which spectra are not restricted to be real, time-reversal symmetry renders the spectra symmetric about the real axis^46^, while particle-hole symmetry makes the spectra symmetric about the imaginary axis^26,33,52^; they are topologically equivalent in the complex energy plane (see Supplementary Note 2 for details). This crucial observation leads to the expectation that non-Hermiticity topologically unifies symmetry classes (Fig. 2), as shown below. We note that the role of chiral symmetry is unchanged in non-Hermitian physics, since it is defined to be unitary and does not involve complex conjugation.

**Table 1:** Constraints on the complex spectra imposed by the Altland–Zirnbauer (AZ) symmetry

AZ symmetry	Hermitian	Non-Hermitian
Time-reversal	No constraints	$E \in {\Bbb R}$ or (*E*, E^*^)
Particle-hole	*E* = 0 or (*E*, −*E*)	$E \in {\mathrm{i}}{\Bbb R}$ or (*E*, −E^*^)
Chiral	*E* = 0 or (*E*, −*E*)	*E* = 0 or (*E*, −*E*)

In Hermitian systems, time-reversal symmetry places no constraints on the real spectra, while particle-hole symmetry gives zero energy *E* = 0 or opposite-sign pairs (*E*, −*E*). In non-Hermitian systems, by contrast, time-reversal symmetry gives real energies $E \in {\Bbb R}$ or complex-conjugate pairs (*E*, E^*^), while particle-hole symmetry gives pure imaginary energies $E \in {\mathrm{i}}{\Bbb R}$ or pairs (*E*, −E^*^). Chiral symmetry gives *E* = 0 or pairs (*E*, −*E*) in both Hermitian and non-Hermitian systems.

### Topological unification

Motivated by the topological equivalence of time-reversal and particle-hole symmetries in the complex energy plane, we consider a general anti-unitary symmetry $\mathcal{A}$ defined by Eq. (1). Here $\mathcal{A}$ reduces to the operator that corresponds to time-reversal (particle-hole) symmetry for *φ* = 0 (*φ* = *π*). Remarkably, only time-reversal and particle-hole symmetries are allowed when *H* is Hermitian. To see this, we take Hermitian conjugation of Eq. (1) and use Hermiticity of *H* $\left( {H^\dagger = H} \right)$ and the definition of anti-unitary symmetry $\left( {{\cal A}^\dagger = {\cal A}^{ - 1}} \right)$. We then obtain ${\cal A}H{\cal A}^{ - 1} = e^{ - {\mathrm{i}}\varphi }H$, which leads to *φ* = 0, *π*. For non-Hermitian *H*, on the other hand, there are no such constraints.

We study the continuous deformations between a system with time-reversal symmetry and a system with particle-hole symmetry in the presence of a complex-energy gap and an anti-unitary symmetry $\mathcal{A}$. Such deformations cannot be performed for Hermitian Hamiltonians since only the discrete values *φ* = 0, *π* are allowed due to Hermiticity; a topological phase with time-reversal symmetry and that with particle-hole symmetry are distinguished in Hermitian physics. Surprisingly, an arbitrary non-Hermitian Hamiltonian *H*_0_ with time-reversal symmetry can be continuously deformed into a Hamiltonian with particle-hole symmetry because *H*_*φ*_: = *e*^−i*φ*/2^*H*_0_ preserves both complex gap and anti-unitary symmetry $\mathcal{A}$ for all *φ*, and *H*_*π*_ has particle-hole symmetry. Therefore, a topological phase with time-reversal symmetry and that with particle-hole symmetry are unified into the same topological class in non-Hermitian physics. The topological unification of anti-unitary symmetries presents a general symmetry principle in non-Hermitian physics that holds regardless of the definition of a complex gap^68^.

### Topological insulator induced by non-Hermiticity

**Fig. 3:**
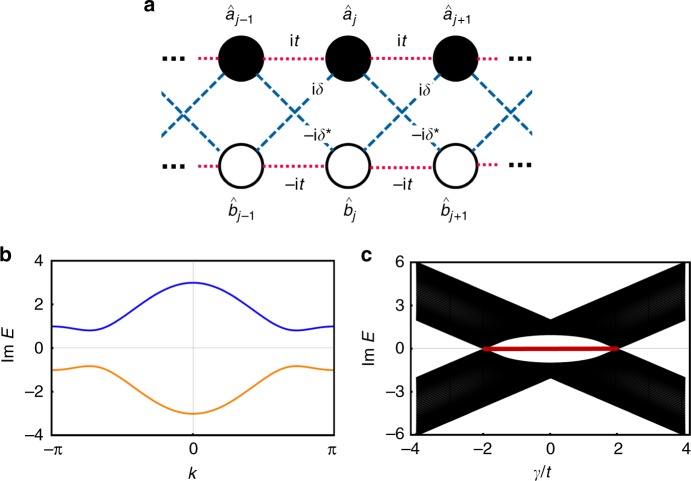
Non-Hermitian one-dimensional topological insulator with time-reversal symmetry. The results shown are for the 1D class AI model described by Eq. (3). **a** Schematic representation of the Hamiltonian. The system consists of the asymmetric hopping and the gain/loss. **b** Energy dispersion of the chain with periodic boundaries (*t* = 1.0, *δ* = 0.5, *γ* = 1.0). The imaginary spectrum is gapped for |*γ*| ≠ 2*t*. **c** Imaginary spectrum of the system of *L* = 50 sites with open boundaries (*t* = 1.0, *δ* = 0.5). Edge states with I*mE* = 0 (red line) emerge in the topological phase (|*γ*/*t*| ≤ 2)

As a consequence of the topological unification of time-reversal and particle-hole symmetries, unique non-Hermitian topological phases emerge that are absent in Hermitian systems. In particular, in accordance with the topological phase in the Majorana chain (1D class D)^14^, non-Hermiticity induces topological phases in one-dimensional insulators that respect time-reversal symmetry with ${\cal T}^2 = + 1$ (1D class AI). Examples include a one-dimensional lattice with two sites per unit cell (Fig. 3a):

3 $$\begin{array}{*{20}{l}} {\hat H_{{\mathrm{NHTI}}}} \hfill & = \hfill & {\mathop {\sum}\limits_j \left\{ {{\mathrm{i}}t\left( {\hat a_{j - 1}^\dagger \hat a_j - \hat b_{j - 1}^\dagger \hat b_j + \hat a_j^\dagger \hat a_{j - 1} - \hat b_j^\dagger \hat b_{j - 1}} \right)} \right.} \hfill \\ {} \hfill & {} \hfill & { + \left[ {{\mathrm{i}}\delta \left( {\hat b_{j - 1}^\dagger \hat a_j - \hat b_{j + 1}^\dagger \hat a_j} \right) + {\mathrm{i}}\delta ^ \ast \left( {\hat a_j^\dagger \hat b_{j - 1} - \hat a_j^\dagger \hat b_{j + 1}} \right)} \right]} \hfill \\ {} \hfill & {} \hfill & { + \left. {{\mathrm{i}}\gamma \left( {\hat a_j^\dagger \hat a_j - \hat b_j^\dagger \hat b_j} \right)} \right\},} \hfill \end{array}$$

where $\hat{a}_j$ $\left( {\hat a_j^\dagger } \right)$ and $\hat b_j$ $\left( {\hat b_j^\dagger } \right)$ denote the annihilation (creation) operators on each sublattice site *j*, *t* > 0 and $\delta \in {\Bbb C}$ are the asymmetric-hopping amplitudes, and $\gamma \in {\Bbb R}$ is the balanced gain and loss. Such gain and loss have been experimentally implemented in various systems^36–42,57–66^ and the asymmetric hopping in optical systems^67^. The system respects time-reversal symmetry $\left( {\hat{\cal T}\hat H_{{\mathrm{NHTI}}}\hat{\cal T}^{ - 1} = \hat H} \right)$, where the time-reversal operation is defined by $\hat{\cal T} \hat a_j \hat{\cal T}^{ - 1} = \hat b_j$, $\hat{\cal T}{\kern 1pt} \hat b_j{\kern 1pt} \hat{\cal T}^{ - 1} = \hat a_j$, and $\hat{\cal T}\,z\,\hat{\cal T}^{ - 1} = z^ \ast$ for all $z \in {\Bbb C}$. The eigenstates form two bands in momentum space, for which the Hamiltonian is determined as $\vec h\left( k \right) \cdot \vec \sigma$ with *h*_*x*_ = −2i Im[*δ*]sin *k*, *h*_*y*_ = = 2i Re [*δ*] sin *k*, *h*_*z*_ = i (*γ* + 2*t* cos *k*), and Pauli matrices $\vec \sigma : = (\sigma _x,\sigma _y,\sigma _z)$. The energy dispersion is obtained as $E_ \pm \left( k \right) = \pm {\mathrm{i}}\sqrt {\left( {\gamma + 2t\,{\mathrm{cos}}\,k} \right)^2 + 4\left| \delta \right|^2{\mathrm{sin}}^2k}$, and hence the complex bands are separated from each other by the energy gap with magnitude min {2|*γ* + 2*t*|, 2|*γ* - 2*t*|} (Fig. 3b).

In parallel with the Majorana chain^14^, the topological invariant ${\nu}_{\mathrm{AI}}$ is defined by

4 $$\left( { - 1} \right)^{\nu _{{\mathrm{AI}}}}: = {\mathrm{sgn}}\left[ {{\mathrm{i}}h_z\left( 0 \right) \cdot {\mathrm{i}}h_z\left( \pi \right)} \right] = - {\mathrm{sgn}}[\gamma ^2 - 4t^2].$$

As a hallmark of the non-Hermitian topological phase, a pair of edge states with zero imaginary energy appears when the bulk has non-trivial topology (*ν*_AI_ = 1; Fig. 3c). Whereas the bulk states that belong to the band *E*_+_ (*E*_*−*_) are amplified (attenuated) with time, the mid-gap edge states are topologically protected from such amplification and attenuation. In the case of *t* = *δ*, for instance, the topologically protected edge states are obtained as

5 $$\begin{array}{l}\hat \Psi _{{\mathrm{edge}}}^{({\mathrm{left}})} \propto {\mathrm{i}}\mathop {\sum}\limits_{j = 1}^L \left( { - \frac{\gamma }{{2t}}} \right)^{j - 1}(\hat a_j - \hat b_j),\\ \hat \Psi _{{\mathrm{edge}}}^{({\mathrm{right}})} \propto \mathop {\sum}\limits_{j = 1}^L \left( { - \frac{\gamma }{{2t}}} \right)^{j - 1}\left( {\hat a_{L - j + 1} + \hat b_{L - j + 1}} \right),\end{array}$$

which satisfy $\left\| {[\hat H_{{\mathrm{NHTI}}},\hat \Psi _{{\mathrm{edge}}}]} \right\| = O{\kern 1pt} (e^{ - L/\xi })$ with the localization length $\xi := - (\log |\gamma/2t|)^{-1}$. These edge states are immune to disorder that respects time-reversal symmetry (see Supplementary Note 7 for details), which is a signature of the topological phase. We emphasize that topological phases are absent in 1D class AI in the presence of Hermiticity^3–5^; non-Hermiticity induces the unique non-equilibrium topological phase as a result of the topological unification of time-reversal and particle-hole symmetries. Whereas the system is an insulator and does not support non-Abelian Majorana fermions, the sublattice degrees of freedom $\hat a_j$ and $\hat b_j$ play the roles of particles and holes in the Majorana chain; the Majorana edge states, which are equal-superposition states of particles and holes, correspond to the equal superposition states of the two sublattices $\hat a_j$ and $\hat b_j$ in the non-Hermitian topological insulator.

### Emergent non-Hermitian topological phases

**Fig. 4:**
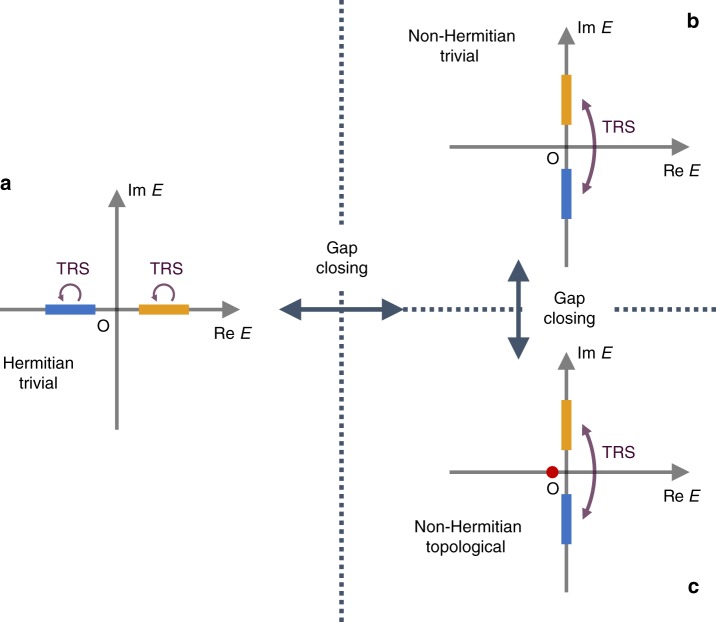
Complex-band structure of non-Hermitian one-dimensional insulators with time-reversal symmetry (1D class AI). Blue and yellow bands represent complex bands and red dots represent topologically protected edge states. **a** Hermitian gapped band structure. All the bands individually respect time-reversal symmetry, and topological phases are absent. **b**, **c** Non-Hermitian gapped band structure in 1D class AI for **b** the trivial phase and **c** the topological phase. Two bands are paired via time-reversal symmetry and the complex-gap closing associated with a topological phase transition should occur between (**a**) and (**b**, **c**)

The topological phases induced by non-Hermiticity are not specific to the above model but general for all the non-Hermitian systems with anti-unitary symmetry. To see this, we examine the complex-band structure of a generic two-band system (*E*_+_ (*k*), *E*_*−*_ (*k*)) in 1D class AI. In the presence of Hermiticity, the real bands individually respect time-reversal symmetry: $E_ \pm \left( k \right) = E_ \pm ^ \ast \left( { - k} \right)$ (Fig. 4a), where topological phases are absent^3,5^. In the presence of strong non-Hermiticity, on the other hand, time-reversal symmetry is spontaneously broken and the complex bands are paired via time-reversal symmetry: $E_ + \left( k \right) = E_ - ^ \ast \left( { - k} \right)$. Importantly, this system has the same band structure as the Hermitian topological superconductor protected by particle-hole symmetry (1D class D) as a direct consequence of the topological unification of time-reversal and particle-hole symmetries; it exhibits both trivial (Fig. 4b) and topological (Fig. 4c) phases according to the ${\Bbb Z}_2$ topological invariant defined by Eq. (4). The latter band structure becomes gapless in the presence of Hermiticity due to *E*_+_ (*k*_0_) = *E*_−_ (*k*_0_) for a time-reversal-invariant momentum *k*_0_ ∈ {0, *π*}.

Remarkably, the emergent non-Hermitian topological phases cannot be continuously deformed into any Hermitian phase that belongs to the same symmetry class. In fact, there should exist a non-Hermitian Hamiltonian that satisfies *E*_+_ (*k*) = *E*_*−*_ (−*k*) between the two types of band structures, and the complex gap closes at *k* = *k*_0_. Thus complex-gap closing associated with a topological phase transition should occur between these phases. We also emphasize that the above discussions are applicable to all the non-Hermitian topological phases in any spatial dimension protected by anti-unitary symmetry. Here the corresponding topological invariants are solely determined by the relationship between symmetry and the complex-band structure as in the Hermitian case^22^.

### Quantum spin Hall insulator

Topological phases survive non-Hermiticity also in two dimensions. In fact, the ${\Bbb Z}_2$ topological invariant $\nu_{\mathrm{AII}}$ can be defined in non-Hermitian two-dimensional insulators that respect both time-reversal and parity (inversion) symmetries just like the Hermitian ones^11^:

6 $$\left( { - 1} \right)^{\nu _{{\mathrm{AII}}}}: = \mathop {\prod}\limits_{{\boldsymbol{k}}_0} \mathop {\prod}\limits_{n: {\mathrm{occupied}}} \pi _n\left( {{\boldsymbol{k}}_0} \right),$$

where ***k***_0_ ∈ {(0, 0), (0, *π*), (*π*, 0), (*π*, *π*)} denotes the time-reversal-invariant and inversion-symmetric momenta in the Brillouin zone, and *π*_*n*_(***k***_0_) ∈ {±1} is the parity eigenvalue of the *n*-th Kramers pair at ***k*** = ***k***_0_. In particular, for four-band insulators such as the Kane–Mele model^8^ and the Bernevig–Hughes–Zhang model^9^, the 4 × 4 Hamiltonian in momentum space that satisfies ${\cal T}{\kern 1pt} {\cal H}\left( {\boldsymbol{k}} \right){\cal T}^{ - 1} = {\cal H}\left( { - {\boldsymbol{k}}} \right)$ and ${\cal P}{\cal H}\left( {\boldsymbol{k}} \right) {\cal P}^{ - 1} = {\cal H}\left( { - {\boldsymbol{k}}} \right)$ is expressed as

7 $${\cal H}_{{\mathrm{QSH}}}\left( {\boldsymbol{k}} \right) = d_0\left( {\boldsymbol{k}} \right)I + \vec d\left( {\boldsymbol{k}} \right) \cdot {\vec{\mathrm \Gamma }} + {\mathrm{i}}\mathop {\sum}\limits_{1 \le i < j \le 5} d_{ij}\left( {\boldsymbol{k}} \right){\mathrm{\Gamma }}_{ij},$$

where the coefficients *d*_*i*_’s and *d*_*ij*_’s are real, Γ_*I*_’s are ${\cal P}{\cal T}$-symmetric five Dirac matrices, and Γ_*ij*_’s are their commutators Γ_*ij*_: = [Γ_*i*_, Γ_*j*_]/2i. We notice that Hermiticity leads to *d*_*ij*_ = 0. Here only Γ_1_ and Γ_*ij*_ (2 ≤ *i* < *j* ≤ 5) are invariant under space inversion when Γ_1_ is chosen as $\mathcal{P}$^11^. Moreover, when the parity and time-reversal operators are given as ${\cal P} = \sigma _z$ and ${\cal T} = {\mathrm{i}}s_y{\cal K}$, the Dirac matrices can be expressed as ${\mathrm{\Gamma }}_1 = \sigma _z( = {\cal P})$, ${\mathrm{\Gamma }}_2 = \sigma _y$, ${\mathrm{\Gamma }}_3 = \sigma _xs_x$, ${\mathrm{\Gamma }}_4 = \sigma _xs_y$, and ${\mathrm{\Gamma }}_5 = \sigma _xs_z$ ^11^. Here *σ*_*i*_’s (*s*_*i*_’s) denote the Pauli matrices that describe the sublattice (spin) degrees of freedom. Since the Hamiltonian at ***k*** = ***k***_0_ is invariant under inversion ${\cal P}{\cal H}\left( {{\boldsymbol{k}}_0} \right){\cal P}^{ - 1} = {\cal H}\left( {{\boldsymbol{k}}_0} \right)$, it reduces to ${\cal H}_{{\mathrm{QSH}}}\left( {{\boldsymbol{k}}_0} \right) = d_{0}\left( {{\boldsymbol{k}}_0} \right)I + d_1\left( {{\boldsymbol{k}}_0} \right) {\mathcal{P}} + {\mathrm{i}}\mathop {\sum}\nolimits_{1 \le i < j \le 5} d_{ij}\left( {{\boldsymbol{k}}_0} \right){\mathrm{\Gamma }}_{ij}$; the parity of a Kramers pair at ***k*** = ***k***_0_ corresponds to the sign of *d*_1_(***k***_0_), and the ${\Bbb Z}_2$ topological invariant defined by Eq. (6) is obtained as $\left( { - 1} \right)^{\nu _{{\mathrm{AII}}}} = \mathop {\prod}\nolimits_{{\boldsymbol{k}}_0} {\mathrm{sgn}}\left[ {d_1\left( {{\boldsymbol{k}}_0} \right)} \right]$ as long as complex bands are gapped an*d d*_1_(***k***_0_) is non-zero.

**Fig. 5:**
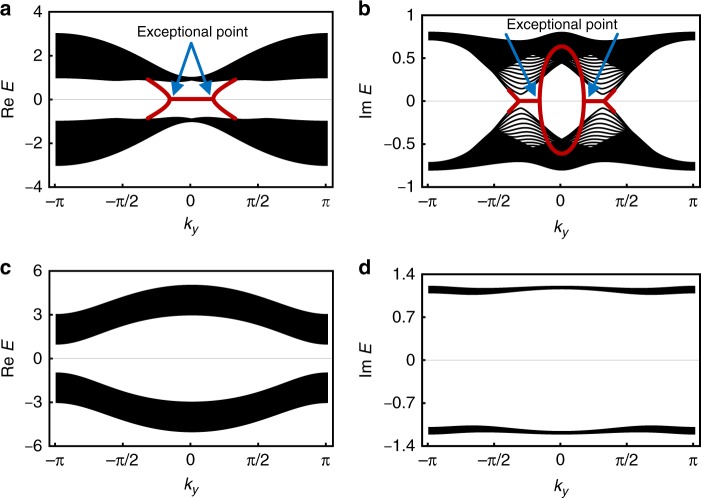
Non-Hermitian quantum spin Hall insulator. The results shown are for the model described by Eq. (7) with *d*_1_(***k***) = *m* + *t* cos *k*_*x*_ + *t* cos *k*_*y*_, *d*_2_(***k***) = *t* sin *k*_*y*_, *d*_3_(***k***) = *λ*(sin *k*_*x*_ + sin *k*_*y*_), *d*_5_(***k***) = *t* sin *k*_*x*_, and *d*_25_(***k***) = *γ* $\left( {t,m,\lambda ,\gamma \in {\Bbb R}} \right)$. We here consider a non-Hermitian two-dimensional insulator on a square lattice with open boundaries in the *x* direction and periodic boundaries in the *y* direction, along which the wavenumber *k*_*y*_ is well-defined. The ${\Bbb Z}_2$ topological invariant is given by $\left( { - 1} \right)^{\nu _{{\mathrm{AII}}}} = {\mathrm{sgn}}{\kern 1pt} [m^2 - 4t^2]$. **a** Real and **b** imaginary parts of the complex spectrum in the topological phase (*t* = 1.0, *m* = -1.0, *λ* = 0.5, *γ* = 0.8; ${\nu}_{\mathrm{AII}}$ = 1). Helical edge states (red curves) appear between the gapped complex bands and form a pair of exceptional points. **c** Real and **d** imaginary parts of the complex spectrum in the trivial phase (*t* = 1.0, *m* = 3.0, *λ* = 0.8, *γ* = 1.2; ${\nu}_{\mathrm{AII}}$ = 0). No gapless states appear between the gapped complex bands

This bulk ${\Bbb Z}_2$ topological invariant corresponds to the emergence of helical edge states (Fig. 5). In stark contrast to Hermitian systems^8–11^, the helical edge states form not a Dirac point but a pair of exceptional points^30,31,40,63,69,70^ and have non-zero imaginary energies at the time-reversal-invariant momenta. Nevertheless, they are immune to disorder due to the generalized Kramers theorem (see Supplementary Notes 5 and 7 for details), which states that all the real parts of energies should be degenerate in the presence of time-reversal symmetry with ${\cal T}^2 = - 1$; the degeneracies of the real parts of energies forbid the continuous annihilation of a pair of helical edge states. Notably, the helical edge states are lasing (see Supplementary Note 8 for details) like chiral edge states in a non-Hermitian Chern insulator^42^.

The topological unification of anti-unitary symmetries indicates that non-Hermitian systems that respect particle-hole symmetry with ${\cal C}^2 = - 1$ (2D class C) also exhibit the ${\Bbb Z}_2$ topological phase, in contrast to the $2{\Bbb Z}$ topological phase in Hermitian physics^3–5^. Here the spin-up and spin-down particles in insulators correspond to particles and holes in superconductors. This emergent ${\Bbb Z}_2$ topological phase is due to the presence of Kramers pairs of particles and holes with imaginary energies, which are forbidden in Hermitian systems where energies are confined to the real axis; non-Hermiticity brings about topological phases unique to non-equilibrium open systems.

## Discussion

Non-Hermiticity manifests itself in many disciplines of physics as gain and loss or asymmetric hopping^43,44^. We have shown that such non-Hermiticity unifies the two fundamental anti-unitary symmetries and consequently topological classification, leading to the prediction of unique non-equilibrium topological phases that are absent at equilibrium. The unveiled topological unification of time-reversal and particle-hole symmetries provides a general symmetry principle in non-Hermitian physics that also justifies a different type of topological classification^68^. The modified topological classification implies that the symmetry unification can bring about physics unique to non-Hermitian systems. It merits further study to explore such unusual properties and functionalities that result from our symmetry principle.

This work has explored topological phases characterized by wave functions in non-Hermitian gapped systems, which is a non-trivial generalization of the Hermitian topological phases. By contrast, non-Hermitian gapless systems possess an intrinsic topological structure, which accompanies exceptional points^69,70^, and has no counterparts in Hermitian systems. This topology can be characterized by a complex-energy dispersion^28,30,31^ and is distinct from the topology defined by wave functions. A complete theory of non-Hermitian topological systems should be formulated on the basis of these two types of topology in a unified manner, which awaits further theoretical development.

## Supplementary information

Supplementary Information

Peer Review File

## Data Availability

The data that support the findings of this study are available from the corresponding author on reasonable request.
