# Supplementary material for: Topological unification of time-reversal and particle-hole symmetries in non-Hermitian physics
Source: Nat Commun. 2019 Jan 17;10:297. doi: 10.1038/s41467-018-08254-y (PMC6336874; doi:10.1038/s41467-018-08254-y)
Supplement: Supplementary file 1 — Supplementary Information [file 41467_2018_8254_MOESM1_ESM.pdf]

Supplementary Information
Topological unification of time-reversal and particle-hole symmetries in non-Hermitian physics

Kawabata *et al.*[*]

[*] kawabata@cat.phys.s.u-tokyo.ac.jp

SUPPLEMENTARY NOTE 1: PHYSICAL MEANING OF A COMPLEX GAP

The presence of a complex gap has a significant influence on the non-equilibrium wave dynamics. To understand this, we consider a three-level system shown in Supplementary Figure 1 (a) as an example. Here the ground state $|g\rangle$ is resonantly coupled to the two excited states $|e_1\rangle$ and $|e_2\rangle$ that have the same energy levels but the different decay rates (linewidths) γ_1 and γ_2 ($\gamma_1 < \gamma_2$), and the driving strength (Rabi frequency) Ω between $|g\rangle$ and $|e_1\rangle$ is equal to that between $|g\rangle$ and $|e_2\rangle$. In the framework of the non-Hermitian band theory, this situation corresponds to the presence of two modes having the same real parts but the different imaginary parts of eigenenergies at a particular wavenumber $\boldsymbol{k}$. Then when the wave function of the system is denoted as $|\psi\rangle$, the wave dynamics (in the rotating frame) is governed by

$$\mathrm{i}\frac{d}{dt}|\psi\rangle = H|\psi\rangle, \quad H := \frac{1}{2}\begin{pmatrix} -\mathrm{i}\gamma_1 & \Omega & 0 \\ \Omega & 0 & \Omega \\ 0 & \Omega & -\mathrm{i}\gamma_2 \end{pmatrix}, \tag{1}$$

where the basis is chosen as $(|e_1\rangle, |g\rangle, |e_2\rangle)$. Here $|\psi\rangle$ represents an electric-field envelope in classical photonics and a quantum wave function in quantum physics (the Planck constant $\hbar$ is set to unity).

Now let us consider driving the system at the Rabi frequency Ω. When the state is initially prepared to be $|g\rangle$, the intensity or the population for the excited state $|e_i\rangle$ ($i = 1, 2$) at time t is obtained as

$$p_i(t) = \left|\langle e_i| e^{-\mathrm{i}Ht} |g\rangle\right|^2. \tag{2}$$

The difference of the decay rates (imaginary parts of the eigenenergies) significantly affects the behavior of the wave dynamics, although the two modes have the same energy levels (real parts of the eigenenergies). In fact, the excitation to the mode with the larger decay rate is suppressed [Supplementary Figure 1 (b)]. We remark that such suppression of the wave dynamics due to the presence of dissipation is known as the quantum Zeno effect [1, 2]. Although termed as the quantum Zeno effect due to some historical reasons, this phenomenon occurs in any linear dynamics of probability amplitudes and especially in classical wave dynamics. In fact, an early experimental demonstration was performed for classical light [3]. These dissipative effects are also relevant in many-body systems [4, 5]. In particular, even a purely imaginary band gap can play a role similar to that of a real band gap and confine the dynamics of a wave packet within the lower band, provided that the driving strength is weak enough compared with the band gap [6].

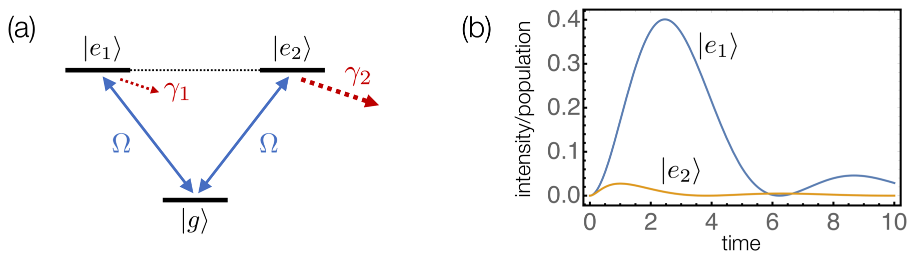

Supplementary Figure 1. Physical meaning of a complex gap. (a) Energy-level diagram of a three-level system. The ground state $|g\rangle$ is resonantly coupled to the two excited states $|e_1\rangle$ and $|e_2\rangle$ that have the same energy levels but the different decay rates (linewidths) γ_1 and γ_2 ($\gamma_1 < \gamma_2$), and the driving strength (Rabi frequency) is equal to Ω for both excited states. (b) Evolution of the intensity or the population for the excited states $|e_1\rangle$ and $|e_2\rangle$ ($\Omega = 1.0$, $\gamma_1 = 0.5$, $\gamma_2 = 5.0$). The excitation to the mode $|e_2\rangle$ is suppressed due to the larger decay rate ($\gamma_1 < \gamma_2$).

SUPPLEMENTARY NOTE 2: SYMMETRY CONSTRAINTS ON COMPLEX SPECTRA

We consider a non-Hermitian Hamiltonian H with a generalized antiunitary symmetry $\mathcal{A}$ defined by $\mathcal{A}H\mathcal{A}^{-1} = e^{\mathrm{i}\varphi}H$ ($0 \leq \varphi < 2\pi$). When $E \in \mathbb{C}$ is an eigenenergy of H and $|\psi\rangle$ is the corresponding eigenstate, we have

$$H(\mathcal{A}|\psi\rangle) = e^{-\mathrm{i}\varphi}\mathcal{A}H|\psi\rangle = e^{-\mathrm{i}\varphi}\mathcal{A}(E|\psi\rangle) = \left(E^* e^{-\mathrm{i}\varphi}\right)(\mathcal{A}|\psi\rangle). \tag{3}$$

Hence $\mathcal{A}|\psi\rangle$ is an eigenstate of H with eigenenergy $E^* e^{-\mathrm{i}\varphi}$. If $|\psi\rangle$ is also an eigenstate of $\mathcal{A}$, we have $E = E^* e^{-\mathrm{i}\varphi}$; otherwise eigenenergies come in $(E,\ E^* e^{-\mathrm{i}\varphi})$ pairs. In particular, when $\mathcal{A}$ describes time-reversal symmetry ($\varphi = 0$), either real eigenenergies or $(E,\ E^*)$ pairs appear [7]; when $\mathcal{A}$ describes particle-hole symmetry ($\varphi = \pi$), either pure imaginary eigenenergies or $(E,\ -E^*)$ pairs appear [8–10].

SUPPLEMENTARY NOTE 3: NON-HERMITIAN MAJORANA CHAIN

Certain symmetry-protected topological phases survive even in the presence of non-Hermiticity. Such phases include superconducting wires that respect particle-hole symmetry with $\mathcal{C}^2 = +1$ (1D class D). Let us express the Bogoliubov-de Gennes (BdG) Hamiltonian as $\mathcal{H}(k) = h_0(k)\, I + \vec{h}(k) \cdot \vec{\sigma}$ with the identity matrix I and Pauli matrices $\vec{\sigma} := (\sigma_x, \sigma_y, \sigma_z)$. Then the presence of particle-hole symmetry $\mathcal{C} := \sigma_x \mathcal{K}$ implies

$$h_{0,x,y}(k) = -h_{0,x,y}^{*}(-k), \quad h_z(k) = h_z^{*}(-k). \tag{4}$$

Therefore, h_z becomes real at the particle-hole-symmetric momentum $k_0 \in \{0, \pi\}$, and hence the topological invariant ν_{D} can be defined by

$$(-1)^{\nu_{\mathrm{D}}} := \mathrm{sgn}\left[h_z(0)\, h_z(\pi)\right] \tag{5}$$

as long as $h_z(0)$ and $h_z(\pi)$ are nonzero. The presence of particle-hole symmetry allows one to introduce the topological invariant ν_{D} as in the Hermitian topological superconductors [11], although $h_{0,x,y}(k_0)$ can be nonzero (pure imaginary) in contrast to Hermitian systems. Moreover, when we introduce Majorana operators $\hat{a}_k$ and $\hat{b}_k$ in momentum space as $\hat{a}_k^{\dagger} = \hat{a}_{-k}$, $\hat{b}_k^{\dagger} = \hat{b}_{-k}$, and $\hat{c}_k = (\hat{a}_k + \mathrm{i}\hat{b}_k)/2$, the Hamiltonian is expressed as

$$\hat{H}_{\mathrm{D}} = \sum_{k \in \mathrm{BZ}} \begin{pmatrix} \hat{c}_k^{\dagger} & \hat{c}_{-k} \end{pmatrix} \begin{pmatrix} h_0 + h_z & h_x - \mathrm{i}h_y \\ h_x + \mathrm{i}h_y & h_0 - h_z \end{pmatrix} \begin{pmatrix} \hat{c}_k \\ \hat{c}_{-k}^{\dagger} \end{pmatrix} = \frac{\mathrm{i}}{2} \sum_{k \in \mathrm{BZ}} \begin{pmatrix} \hat{a}_k^{\dagger} & \hat{b}_k^{\dagger} \end{pmatrix} \begin{pmatrix} -\mathrm{i}h_0 - \mathrm{i}h_x & \mathrm{i}h_y + h_z \\ \mathrm{i}h_y - h_z & -\mathrm{i}h_0 + \mathrm{i}h_x \end{pmatrix} \begin{pmatrix} \hat{a}_k \\ \hat{b}_k \end{pmatrix}. \tag{6}$$

Thus, the non-Hermitian part of the momentum-space Hamiltonian at the particle-hole-symmetric momentum k_0 in the Majorana basis is obtained as $X(k_0) = \mathrm{i}h_z(k_0)\, \sigma_y$, and the topological invariant defined above is also expressed as $(-1)^{\nu_{\mathrm{D}}} = \mathrm{sgn}\left[\mathrm{Pf}\, X(0) \cdot \mathrm{Pf}\, X(\pi)\right]$.

The above discussion applies to a non-Hermitian Majorana chain with asymmetric hopping:

$$\hat{H}_{\mathrm{M}} = \sum_j \left[-t_L \hat{c}_j^{\dagger} \hat{c}_{j+1} - t_R \hat{c}_{j+1}^{\dagger} \hat{c}_j + \Delta \hat{c}_j \hat{c}_{j+1} + \Delta^{*} \hat{c}_{j+1}^{\dagger} \hat{c}_j^{\dagger} - \mu \left(\hat{c}_j^{\dagger} \hat{c}_j - \frac{1}{2} \right) \right], \tag{7}$$

where $\hat{c}_j$ ($\hat{c}_j^{\dagger}$) annihilates (creates) a fermion on site j; $t_L > 0$ ($t_R > 0$) is the hopping amplitude from right to left (from left to right), $\Delta \in \mathbb{C}$ is the p-wave pairing gap, and $\mu \in \mathbb{R}$ is the chemical potential. Here non-Hermiticity arises from the asymmetric hopping $t_L \neq t_R$ [8, 12, 13]. The BdG Hamiltonian is given by

$$h_0 = \mathrm{i}(t_L - t_R) \sin k, \; h_x = 2\, \mathrm{Im}[\Delta] \sin k, \; h_y = -2\, \mathrm{Re}[\Delta] \sin k, \; h_z = \mu + (t_L + t_R) \cos k. \tag{8}$$

Hence the energy dispersion is obtained as

$$E_{\pm}(k) = \mathrm{i}(t_L - t_R) \sin k \pm \sqrt{\left(\mu + (t_L + t_R)\cos k\right)^2 + 4\left|\Delta\right| \sin^2 k}, \tag{9}$$

and the complex bands are separated from each other by the gap of magnitude $\min\left\{2\left|\mu + (t_L + t_R)\right|, \, 2\left|\mu - (t_L + t_R)\right|\right\}$ as schematically illustrated in Supplementary Figure 2 (a,b). Moreover, the topological invariant is obtained as $(-1)^{\nu_{\mathrm{D}}} = \mathrm{sgn}\left[\mu^2 - (t_L + t_R)^2\right]$, and the non-trivial topology of the bulk ($\nu_{\mathrm{D}} = 1$; $|\mu| < t_L + t_R$) is accompanied by the emergence of the Majorana edge states that are topologically protected with particle-hole symmetry as shown in Supplementary Figure 2 (c,d). Therefore, the topological phase in the Majorana chain survives non-Hermiticity.

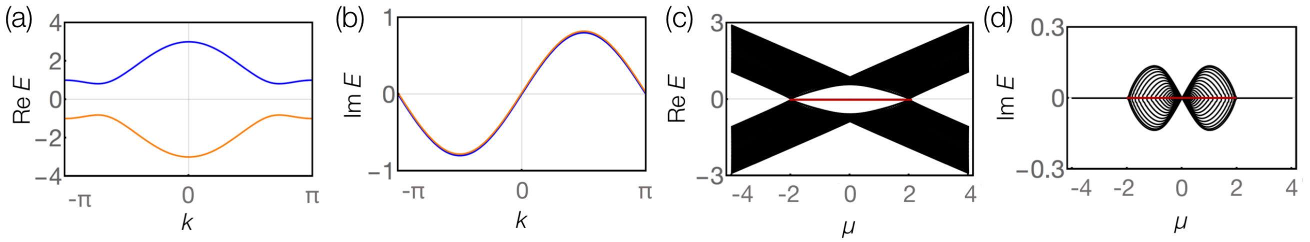

Supplementary Figure 2. Non-Hermitian Majorana chain with asymmetric hopping (1D class D). (a) Real and (b) imaginary parts of the energy dispersion of the chain with periodic boundaries ($t_L = 1.4$, $t_R = 0.6$, $\Delta = 0.5$, $\mu = 1.0$). The complex bands are separated from each other by the gap for $|\mu| \neq t_L + t_R$. (c) Real and (d) imaginary parts of the complex spectrum of the chain with 50 sites and open boundaries ($t_L = 1.4$, $t_R = 0.6$, $\Delta = 0.5$). Majorana edge states with zero energy (red lines) appear in the topological phase ($|\mu| < t_L + t_R = 2.0$).

SUPPLEMENTARY NOTE 4: SYMMETRY AND COMPLEX-BAND STRUCTURE

We consider in detail a complex-band structure of a generic one-dimensional system with time-reversal symmetry (1D class AI). We here investigate a two-band system $(E_+(k), E_-(k))$ for the sake of simplicity, but the discussion can be straightforwardly generalized to arbitrary $2n$-band systems. In the presence of Hermiticity, the real bands individually respect time-reversal symmetry [Supplementary Figure 3 (a)]:

$$E_+(k) = E_+^*(-k), \quad E_-(k) = E_-^*(-k), \tag{10}$$

where topological phases are absent. In the presence of strong non-Hermiticity, on the other hand, time-reversal symmetry is spontaneously broken and the two complex bands are paired via time-reversal symmetry [Supplementary Figure 3 (d, e)]:

$$E_+(k) = E_-^*(-k). \tag{11}$$

Remarkably, this band structure becomes gapless in the presence of Hermiticity since $E_+(k_0) = E_-(k_0)$ holds for a time-reversal-invariant momentum $k_0 \in \{0, \pi\}$, but a complex gap can be open for a non-Hermitian Hamiltonian. This complex-band structure can exhibit both trivial [Supplementary Figure 3 (d)] and topological [Supplementary Figure 3 (e)] phases. Between these two types of band structures, there should exist a non-Hermitian Hamiltonian that satisfies both Supplementary Equation (10) and Supplementary Equation (11) and

$$E_+(k) = E_-(-k). \tag{12}$$

Hence the complex gap closes at a time-reversal-invariant momentum k_0 for this Hamiltonian and gap closing associated with a topological phase transition should be accompanied between these phases [Supplementary Figure 3 (a, d, e)]. Therefore,

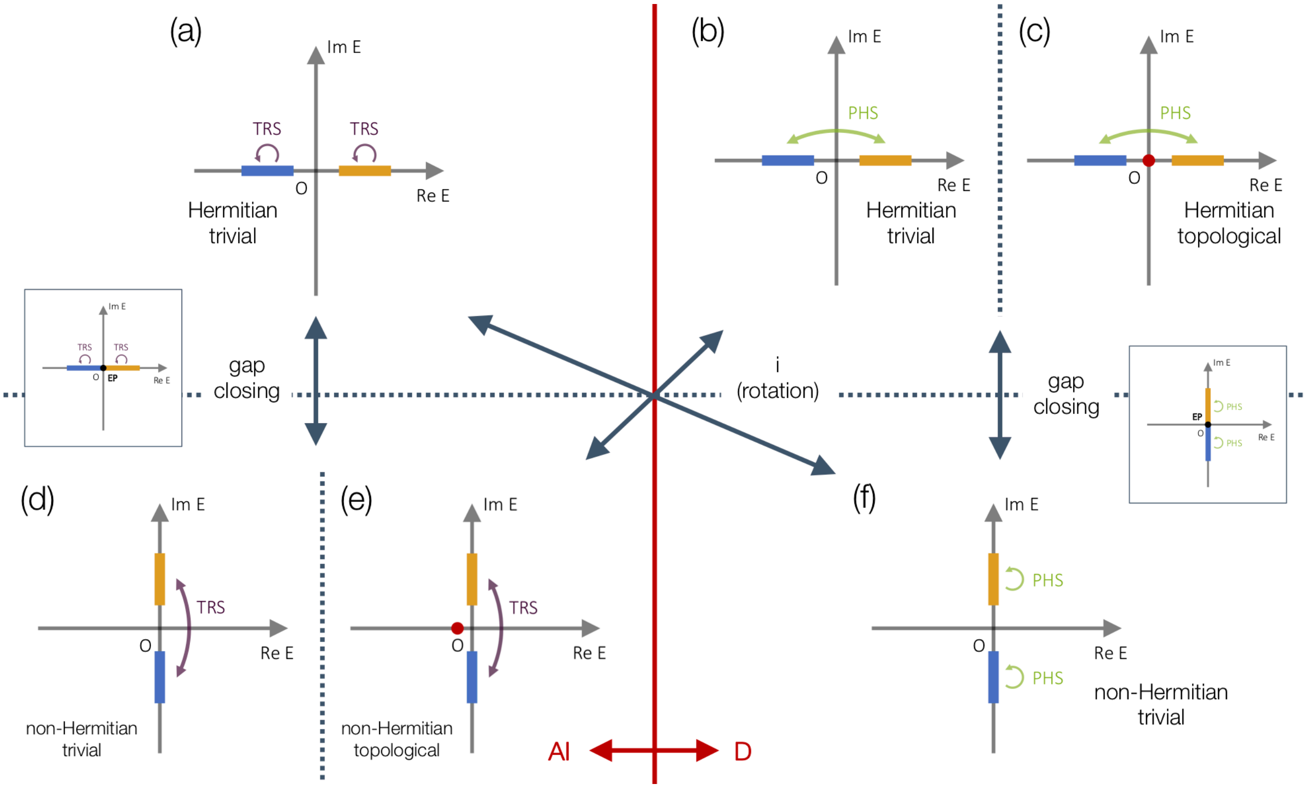

Supplementary Figure 3. Complex-band structure of one-dimensional systems with time-reversal or particle-hole symmetry (1D class AI or D). Blue and yellow bands represent complex bands and red dots represent the topologically protected edge states. When the system respects time-reversal (particle-hole) symmetry, it belongs to 1D class AI; (a, d, e) [D; (b, e, f)]. (a) Hermitian gapped band structure in 1D class AI. All the bands individually respect time-reversal symmetry, and topological phases are absent. (b, c) Hermitian gapped band structure in 1D class D for the (b) trivial and (c) topological phases. Two bands are paired via particle-hole symmetry. (d, e) Non-Hermitian gapped band structure in 1D class AI for the (d) trivial and (e) topological phases. Two bands are paired via time-reversal symmetry and gap closing associated with a topological phase transition should occur between (a) and (d, e). (f) Non-Hermitian gapped band structure in 1D class D. All the bands individually respect particle-hole symmetry, and topological phases are absent.

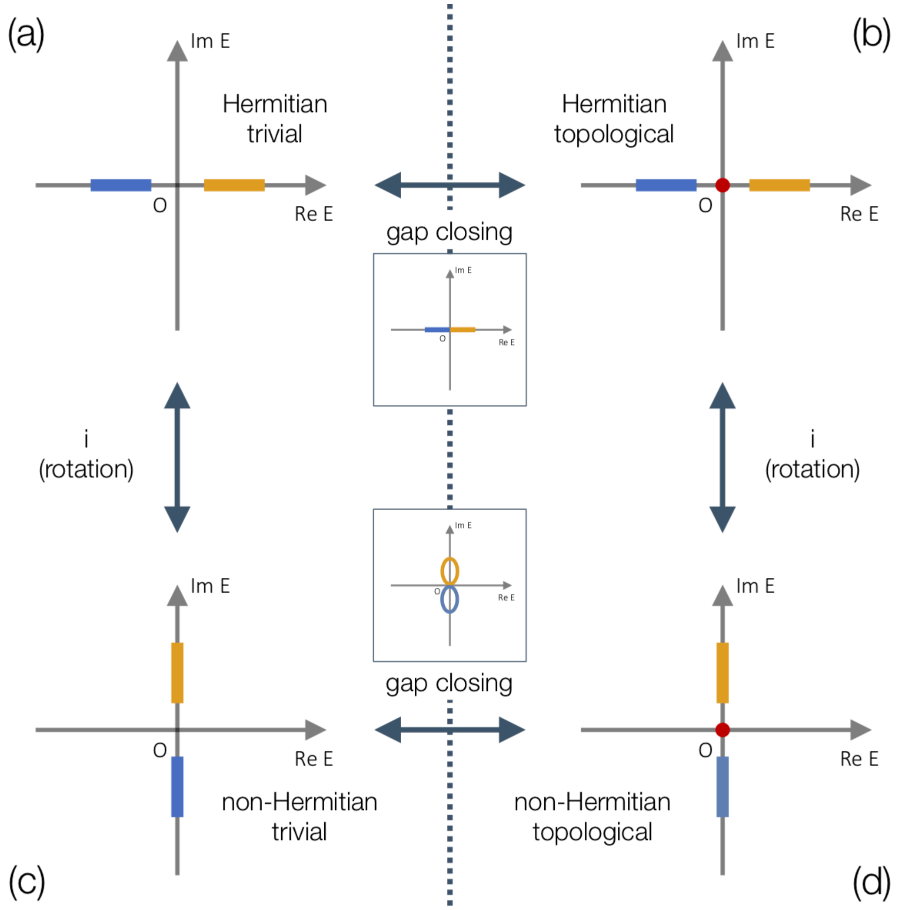

Supplementary Figure 4. Complex-band structure of one-dimensional systems with chiral symmetry (1D class AIII). Blue and yellow bands represent complex bands that are related to each other via chiral symmetry, and red dots represent the topologically protected states with zero energy. (a) Hermitian and trivial gapped band structure. (b) Hermitian and topological band structure. Between (a) and (b), gap closing associated with a topological phase transition should occur. (c) Non-Hermitian and trivial gapped band structure obtained by multiplying the Hamiltonian in (a) by i (rotating the whole complex bands through 90 degrees). (d) Non-Hermitian and topological gapped band structure. Between (c) and (d), gap closing associated with a topological phase transition should occur. Here (b) and (d) can be continuously deformed to each other.

the emergent non-Hermitian topological phases cannot be continuously deformed into any Hermitian phase that belongs to the same symmetry class. Importantly, there is a fundamental and non-trivial relationship between symmetry classes AI and D as a direct consequence of the topological unification of time-reversal and particle-hole symmetries. If we begin with a Hermitian Hamiltonian that belongs to 1D class D and possesses the topological phase [Supplementary Figure 3 (c)] and multiply it by i, we obtain the non-Hermitian Hamiltonian that belongs to 1D class AI and also possesses the topological phase [Supplementary Figure 3 (e)] (notice that the eigenstates remain the same under multiplication by i). The obtained topological phase can be continuously deformed into the emergent non-Hermitian topological phases in 1D class AI.

There is a crucial distinction between this emergent non-Hermitian topological insulator and the non-Hermitian topological insulator protected by chiral symmetry including the Su-Schrieffer-Heeger (SSH) model. In fact, the topological phase in the non-Hermitian SSH model can be continuously deformed into the topological phase in the Hermitian SSH model (Supplementary Figure 4), whereas those in the topological insulator induced by non-Hermiticity cannot be continuously deformed into any Hermitian phase as discussed above. Here the crucial distinction is that chiral symmetry is unitary (unrelated to complex conjugation) in stark contrast to the anti-unitary time-reversal and particle-hole symmetries (see Table I in the main text).

SUPPLEMENTARY NOTE 5: GENERALIZED KRAMERS THEOREM

Anti-unitarity of $\mathcal{A}$ gives $\langle \psi | \mathcal{A} \psi \rangle = \langle \mathcal{A}^2 \psi | \mathcal{A} \psi \rangle$. Assuming $\mathcal{A}^2 = -1$, we obtain $\langle \psi | \mathcal{A} \psi \rangle = - \langle \psi | \mathcal{A} \psi \rangle$, which leads to $\langle \psi | \mathcal{A} \psi \rangle = 0$. If $| \psi \rangle$ and $\mathcal{A} | \psi \rangle$ belong to the same eigenenergy ($E = E^* e^{-i\varphi}$), they are orthogonal and thus degenerate.

When $\mathcal{A}$ describes time-reversal symmetry ($\varphi = 0$), this reduces to the conventional Kramers degeneracy; when $\mathcal{A}$ describes particle-hole symmetry ($\varphi = \pi$), this implies the degeneracy with pure imaginary eigenenergies.

SUPPLEMENTARY NOTE 6: NON-HERMITIAN QUANTUM SPIN HALL INSULATOR

A generic non-Hermitian Hamiltonian that forms four bands in momentum space can be expressed by the identity matrix I, five Dirac matrices Γ_i, and their ten commutators $\Gamma_{ij} := [\Gamma_i, \Gamma_j]/2\mathrm{i}$ as in Eq. (7) in the main text. If we choose the Dirac matrices to be $\mathcal{PT}$-symmetric $[(\mathcal{PT})\,\Gamma_i\,(\mathcal{PT})^{-1} = \Gamma_i]$, their commutators become anti-$\mathcal{PT}$-symmetric $[(\mathcal{PT})\,\Gamma_{ij}\,(\mathcal{PT})^{-1} = -\Gamma_{ij}]$ due to the anti-unitarity of $\mathcal{T}$. In the simultaneous presence of time-reversal and inversion symmetries, the Hamiltonian is also $\mathcal{PT}$-symmetric $[(\mathcal{PT})\,\mathcal{H}_{\mathrm{QSH}}(\boldsymbol{k})\,(\mathcal{PT})^{-1} = \mathcal{H}_{\mathrm{QSH}}(\boldsymbol{k})]$, which makes the coefficients $d_0(\boldsymbol{k})$, $d_i(\boldsymbol{k})$, and $d_{ij}(\boldsymbol{k})$ real. Moreover, if we choose Γ_1 as $\mathcal{P}$, we have [14]

$$\mathcal{P}\,\Gamma_i\,\mathcal{P}^{-1} = \begin{cases} +\Gamma_1 & \text{for } i = 1, \\ -\Gamma_i & \text{for } i \neq 1, \end{cases} \qquad \mathcal{T}\,\Gamma_i\,\mathcal{T}^{-1} = \begin{cases} +\Gamma_1 & \text{for } i = 1, \\ -\Gamma_i & \text{for } i \neq 1; \end{cases}$$
$$\mathcal{P}\,\Gamma_{ij}\,\mathcal{P}^{-1} = \begin{cases} -\Gamma_{1j} & \text{for } i = 1, \\ +\Gamma_{ij} & \text{for } i \neq 1, \end{cases} \qquad \mathcal{T}\,\Gamma_{ij}\,\mathcal{T}^{-1} = \begin{cases} +\Gamma_{1j} & \text{for } i = 1, \\ -\Gamma_{ij} & \text{for } i \neq 1. \end{cases} \tag{13}$$

Therefore, the presence of both time-reversal and inversion symmetries $[\mathcal{T}\,\mathcal{H}_{\mathrm{QSH}}(\boldsymbol{k})\,\mathcal{T}^{-1} = \mathcal{H}_{\mathrm{QSH}}(-\boldsymbol{k})$ and $\mathcal{P}\,\mathcal{H}_{\mathrm{QSH}}(\boldsymbol{k})\,\mathcal{P}^{-1} = \mathcal{H}_{\mathrm{QSH}}(-\boldsymbol{k})]$ implies

$$d_i(-\boldsymbol{k}) = \begin{cases} +d_i(\boldsymbol{k}) & \text{for } i = 0, 1, \\ -d_i(\boldsymbol{k}) & \text{for } i \geq 2; \end{cases} \qquad d_{ij}(-\boldsymbol{k}) = \begin{cases} -d_{1j}(\boldsymbol{k}) & \text{for } i = 1, \\ +d_{ij}(\boldsymbol{k}) & \text{for } i \geq 2. \end{cases} \tag{14}$$

Hence d_i's for $i \geq 2$ and d_{1j}'s vanish at the time-reversal-invariant and inversion-symmetric momenta $\boldsymbol{k} = \boldsymbol{k}_0$.

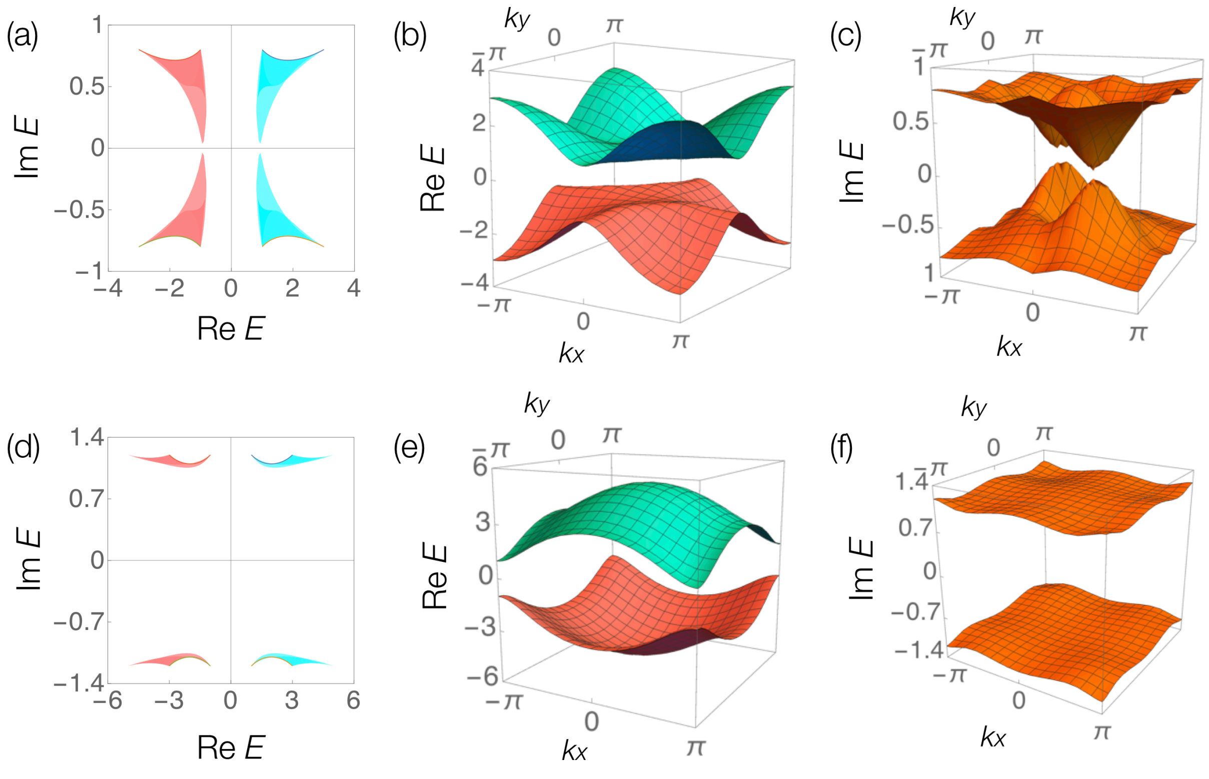

Supplementary Figure 5. Complex-band structures of the non-Hermitian quantum spin Hall insulator (2D class AII). Cyan (pink) bands correspond to $E(k)$, $E^*(k)$ $[-E(k), -E^*(k)]$ in (a, b, d, e). (a) Gapped bands in the complex energy plane $(\mathrm{Re}\,E,\ \mathrm{Im}\,E)$, and (b) real and (c) imaginary parts of the energy dispersion as a function of the wavenumber $(k_x,\ k_y)$ in the topological phase ($t = 1.0$, $m = -1.0$, $\lambda = 0.5$, $\gamma = 0.8$; $\nu_{\mathrm{AII}} = 1$). (d) Gapped bands in the complex energy plane $(\mathrm{Re}\,E,\ \mathrm{Im}\,E)$, and (e) real and (f) imaginary parts of the energy dispersion as a function of the wavenumber $(k_x,\ k_y)$ in the trivial phase ($t = 1.0$, $m = 3.0$, $\lambda = 0.8$, $\gamma = 1.2$; $\nu_{\mathrm{AII}} = 0$).

In particular, we consider the non-Hermitian quantum spin Hall insulator described by Eq. (7) in the main text with

$$d_1(\boldsymbol{k}) = m + t\cos k_x + t\cos k_y,\ d_2(\boldsymbol{k}) = t\sin k_y,\ d_3(\boldsymbol{k}) = \lambda(\sin k_x + \sin k_y),\ d_5(\boldsymbol{k}) = t\sin k_x,\ d_{25}(\boldsymbol{k}) = \gamma, \tag{15}$$

where t, m, λ, and γ are real. The eigenstates form four bands in momentum space and their energy dispersions are obtained as $(E(k), E^*(k), -E(k), -E^*(k))$. Using the anticommutation relations $\{\Gamma_i, \Gamma_j\} = 0$, $\{\Gamma_1, \Gamma_{25}\} = -2\Gamma_{34}$, $\{\Gamma_3, \Gamma_{25}\} = 2\Gamma_{14}$, and $\{\Gamma_2, \Gamma_{25}\} = \{\Gamma_4, \Gamma_{25}\} = 0$, we have

$$\begin{aligned}\mathcal{H}^2_{\mathrm{QSH}}(\boldsymbol{k}) = &\left[(m + t\cos k_x + t\cos k_y)^2 + t^2\sin^2 k_x + t^2\sin^2 k_y + \lambda^2(\sin k_x + \sin k_y)^2 - \gamma^2\right] I \\ &- 2\mathrm{i}\gamma(m + t\cos k_x + t\cos k_y)\Gamma_{34} + 2\mathrm{i}\gamma\lambda(\sin k_x + \sin k_y)\Gamma_{14},\end{aligned} \tag{16}$$

which leads to

$$\begin{aligned}E(k) = &\Big\{(m + t\cos k_x + t\cos k_y)^2 + t^2\sin^2 k_x + t^2\sin^2 k_y + \lambda^2(\sin k_x + \sin k_y)^2 - \gamma^2 \\ &+2\mathrm{i}\gamma\left[(m + t\cos k_x + t\cos k_y)^2 + \lambda^2(\sin k_x + \sin k_y)\right]^{1/2}\Big\}^{1/2}.\end{aligned} \tag{17}$$

The complex bands are gapped with the same parameters used in Fig. 5 in the main text (Supplementary Figure 5). The $\mathbb{Z}_2$ topological invariant is determined as

$$(-1)^{\nu_{\mathrm{AII}}} = \mathrm{sgn}\left[d_1(0,0)\,d_1(0,\pi)\,d_1(\pi,0)\,d_1(\pi,\pi)\right] = \mathrm{sgn}\left[m^2 - 4t^2\right]. \tag{18}$$

SUPPLEMENTARY NOTE 7: ROBUSTNESS AGAINST DISORDER

The topological phase in the non-Hermitian Majorana chain given by Supplementary Equation (7) is immune to disorder that respects particle-hole symmetry. To confirm this robustness, we consider the following disordered non-Hermitian Majorana chain:

$$\hat{H} = \sum_j \left[-(t_L)_j\,\hat{c}_j^\dagger\hat{c}_{j+1} - (t_R)_j\,\hat{c}_{j+1}^\dagger\hat{c}_j + \Delta\hat{c}_j\hat{c}_{j+1} + \Delta^*\hat{c}_{j+1}^\dagger\hat{c}_j^\dagger - \mu_j\left(\hat{c}_j^\dagger\hat{c}_j - \frac{1}{2}\right)\right], \tag{19}$$

where $(t_L)_j$ $[(t_R)_j]$ is the disordered hopping amplitude from site $j+1$ to j (from site j to $j+1$), and μ_j is the disordered chemical potential on site j. The Majorana edge states are protected to have zero energy even in the presence of disorder (Supplementary Figure 6); they are topologically protected with particle-hole symmetry.

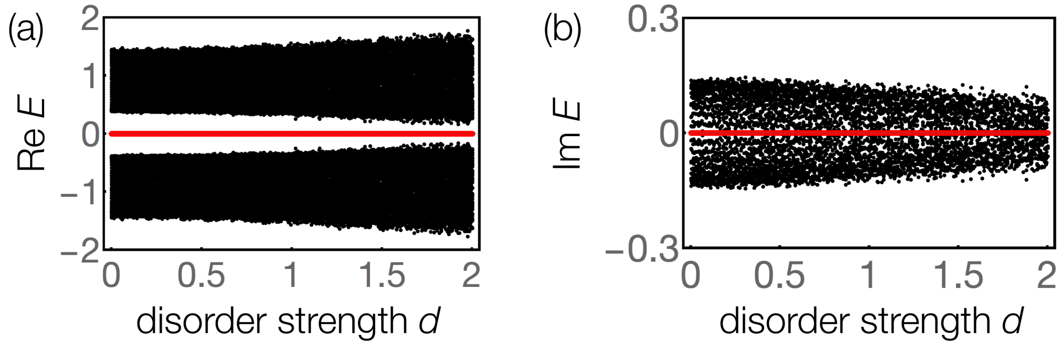

Supplementary Figure 6. Robustness against disorder in the non-Hermitian Majorana chain. (a) Real and (b) imaginary parts of the complex spectrum of the disordered Majorana chain described by Supplementary Equation (19) as a function of the disorder strength d. Black and red dots represent the bulk and Majorana edge states, respectively. The chain with 50 sites is characterized by the parameters $(t_L)_j = 1.4 + 0.3\epsilon_j$, $(t_R)_j = 0.6 + 0.3\epsilon'_j$, $\Delta = 0.5$, and $\mu_j = 1.0 + d\epsilon''_j$, where $(t_L)_j$ $[(t_R)_j]$ is the disordered hopping amplitude from site $j+1$ to j (from site j to $j+1$), μ_j is the disordered chemical potential on site j, and ϵ_j, ϵ'_j and ϵ''_j are random variables uniformly distributed over $[-0.5, 0.5]$.

The topological phase in the non-Hermitian topological insulator given by Eq. (3) in the main text is also immune to disorder that respects time-reversal symmetry. To confirm this robustness, we consider the following disordered non-Hermitian chain:

$$\hat{H} = \sum_j \Big[\mathrm{i} t_j \left(\hat{a}_{j-1}^{\dagger} \hat{a}_j - \hat{b}_{j-1}^{\dagger} \hat{b}_j + \hat{a}_j^{\dagger} \hat{a}_{j-1} - \hat{b}_j^{\dagger} \hat{b}_{j-1} \right) + \left(\mathrm{i}\delta_j \left(\hat{b}_{j-1}^{\dagger} \hat{a}_j - \hat{b}_{j+1}^{\dagger} \hat{a}_j \right) + \mathrm{i}\delta_j^{*} \left(\hat{a}_j^{\dagger} \hat{b}_{j-1} - \hat{a}_j^{\dagger} \hat{b}_{j+1} \right) \right) + \mathrm{i}\gamma_j \left(\hat{a}_j^{\dagger} \hat{a}_j - \hat{b}_j^{\dagger} \hat{b}_j \right) \Big], \tag{20}$$

where t_j and δ_j are the disordered hopping amplitudes between sites $j-1$ and j, and γ_j is the disordered gain/loss on site j. The edge states are protected to have zero energy even in the presence of disorder (Supplementary Figure 7); they are topologically protected with time-reversal symmetry.

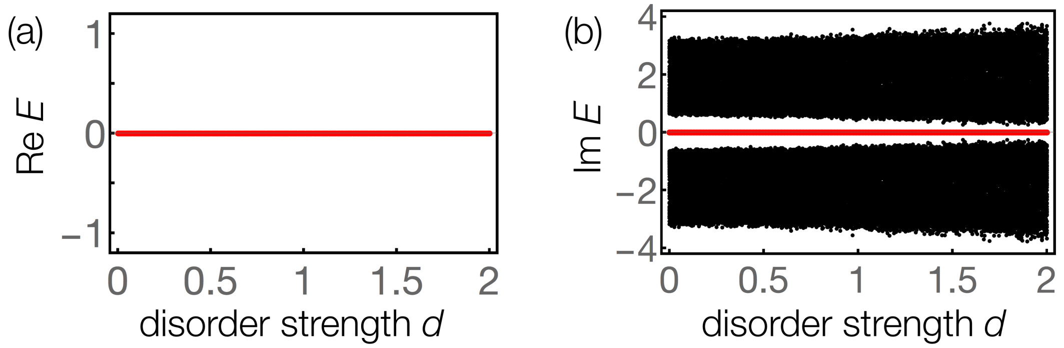

Supplementary Figure 7. Robustness against disorder in the non-Hermitian topological insulator protected by time-reversal symmetry. (a) Real and (b) imaginary parts of the complex spectrum of the disordered non-Hermitian topological insulator described by Supplementary Equation (20) as a function of the disorder strength d. Black and red dots represent the bulk and edge states, respectively. The insulator with 50 sites is characterized by the parameters $t_j = 1.0 + 0.5\,\epsilon_j$, $\delta_j = 0.5 + 0.3\,\epsilon_j'$, and $\gamma_j = 1.0 + d\,\epsilon_j''$, where t_j and δ_j are the disordered hopping amplitudes between sites $j-1$ and j, γ_j is the disordered gain/loss on site j, and ϵ_j, ϵ_j', and ϵ_j'' are random variables uniformly distributed over $[-0.5,\,0.5]$.

Finally, the topological phase in the non-Hermitian quantum spin Hall insulator given by Eq. (7) and shown in Fig. 5 in the main text is immune to disorder that respects time-reversal symmetry. To confirm this robustness, we consider the following disordered non-Hermitian insulator with open boundaries in the x direction and periodic boundaries in the y direction:

$$\hat{H} = \sum_x \sum_{k_y \in \mathrm{BZ}} \Big\{ \frac{1}{2} \left[\hat{\vec{c}}_{x,\,k_y}^{\,\dagger} \left(t \left(\sigma_z \otimes I - \mathrm{i}\sigma_x \otimes s_z \right) - \mathrm{i}\lambda_x \sigma_x \otimes s_x \right) \hat{\vec{c}}_{x+1,\,k_y} + \mathrm{H.c.} \right] + \hat{\vec{c}}_{x,\,k_y}^{\,\dagger} \left[\left(m_x + t\cos k_y \right) \sigma_z \otimes I + \left(t \sin k_y \right) \sigma_y \otimes I + \left(\lambda_x \sin k_y \right) \sigma_x \otimes s_x - \mathrm{i}\gamma_x \sigma_z \otimes s_z \right] \hat{\vec{c}}_{x,\,k_y} \Big\}, \tag{21}$$

where $\hat{\vec{c}}_{x,\,k_y}$ ($\hat{\vec{c}}_{x,\,k_y}^{\,\dagger}$) annihilates (creates) a fermion on site x and with momentum k_y that has four internal degrees of freedom; m_x, λ_x, and γ_x denote the disordered parameters. The helical edge states are topologically protected with time-reversal symmetry even in the presence of disorder [Supplementary Figure 8 (a,b)]. Moreover, we confirm their robustness also in the following disordered non-Hermitian insulator with open boundaries in both x and y directions:

$$\hat{H} = \sum_x \sum_y \Big\{ \frac{1}{2} \left[\hat{\vec{c}}_{x,\,y}^{\,\dagger} T_{x,\,y}^{(x)} \hat{\vec{c}}_{x+1,\,y} + \hat{\vec{c}}_{x,\,y}^{\,\dagger} T_{x,\,y}^{(y)} \hat{\vec{c}}_{x,\,y+1} + \mathrm{H.c.} \right] + \hat{\vec{c}}_{x,\,y}^{\,\dagger} M_{x,\,y} \hat{\vec{c}}_{x,\,y} \Big\}, \tag{22}$$

with

$$\begin{pmatrix} T_{x,\,y}^{(x)} \\ T_{x,\,y}^{(y)} \end{pmatrix} := \begin{pmatrix} t \left(\sigma_z \otimes I - \mathrm{i}\sigma_x \otimes s_z \right) - \mathrm{i}\lambda_{x,y}\, \sigma_x \otimes s_x \\ t \left(\sigma_z \otimes I - \mathrm{i}\sigma_y \otimes I \right) - \mathrm{i}\lambda_{x,y}\, \sigma_x \otimes s_x \end{pmatrix}, \quad M_{x,\,y} := m_{x,y}\, \sigma_z \otimes I - \mathrm{i}\gamma_{x,y}\, \sigma_z \otimes s_z. \tag{23}$$

Here $\hat{\vec{c}}_{x,\,y}$ ($\hat{\vec{c}}_{x,\,y}^{\,\dagger}$) annihilates (creates) a fermion on site $(x,\,y)$, and $m_{x,y}$, $\lambda_{x,y}$, and $\gamma_{x,y}$ denote the disordered parameters. The helical edge states are robust also in this case [Supplementary Figure 8 (c)]; the topological phase in the non-Hermitian quantum spin Hall insulator is immune to disorder that respects time-reversal symmetry.

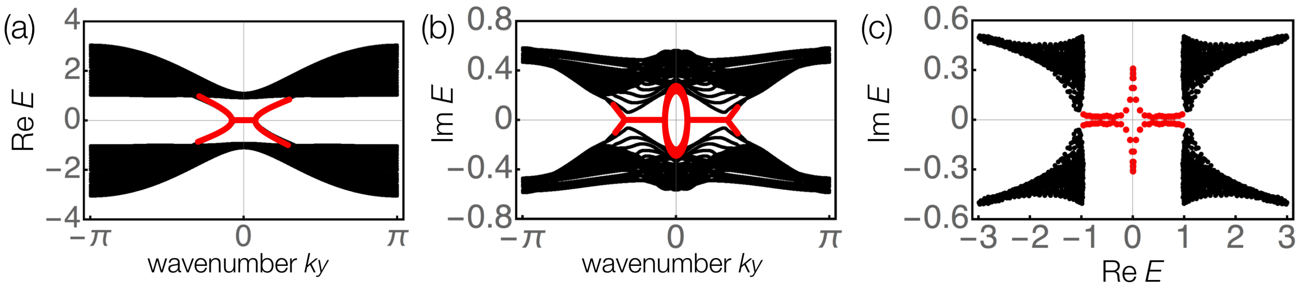

Supplementary Figure 8. Robustness against disorder in the non-Hermitian quantum spin Hall insulator. (a) Real and (b) imaginary parts of the complex spectrum of the disordered non-Hermitian quantum spin Hall insulator described by Supplementary Equation (21) as a function of the wavenumber k_y. Black and red dots represent the bulk and edge states, respectively. The open boundary condition is imposed in the x direction, whereas the periodic boundary condition is imposed in the y direction. The insulator with 30 sites is characterized by the parameters $t = 1.0$, $m_x = -1.0 + 0.3\,\epsilon_x$, $\lambda_x = 0.5 + 0.2\,\epsilon'_x$and $\gamma_x = 0.5 + 0.3\,\epsilon''_x$, where ϵ_x, ϵ'_x, and ϵ''_x are random variables uniformly distributed over $[-0.5,\ 0.5]$. (c) Complex spectrum of the disordered non-Hermitian quantum spin Hall insulator described by Supplementary Equation (22) and Supplementary Equation (23). The open boundary condition is imposed in both x and y directions. The insulator with 30×30 sites is characterized by the parameters $t = 1.0$, $m_{x,y} = -1.0 + 0.3\,\epsilon_{x,y}$, $\lambda_{x,y} = 0.5 + 0.2\,\epsilon'_{x,y}$ and $\gamma_{x,y} = 0.5 + 0.3\,\epsilon''_{x,y}$.

SUPPLEMENTARY NOTE 8: DYNAMICS OF TOPOLOGICAL EDGE STATES IN NON-HERMITIAN SYSTEMS: EXPERIMENTAL SIGNATURE OF NON-HERMITIAN TOPOLOGICAL PHASES

A hallmark of the topological phases is the emergence of robust edge states that reflect the non-trivial topology of the bulk. Remarkably, non-Hermiticity can make these edge states amplified (lasing) [15–18] and anomalous [19]. The non-Hermitian topological insulators considered in this work also exhibit unique topologically protected edge states that have no Hermitian counterparts, which serves as an experimental signature of the non-Hermitian topological phases. In the following, we consider

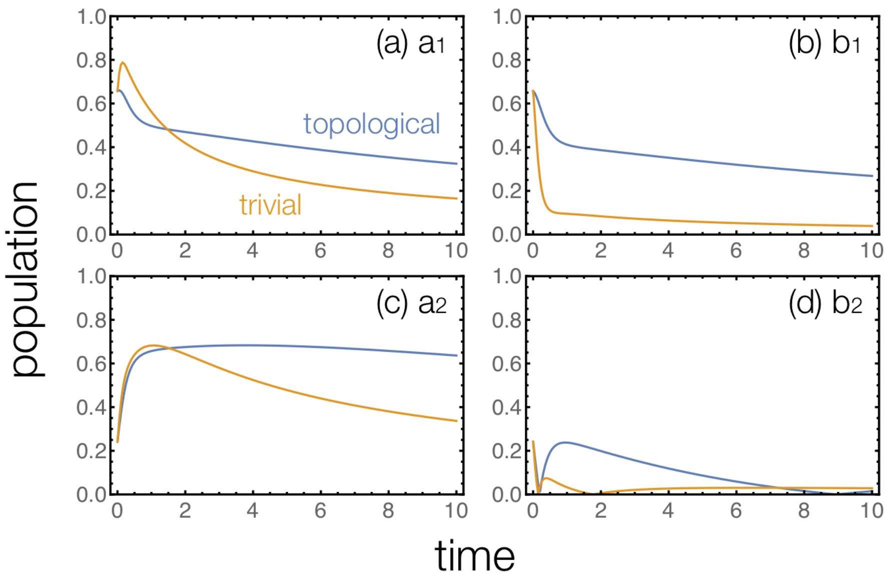

Supplementary Figure 9. Population dynamics in the non-Hermitian topological insulator protected by time-reversal symmetry (1D class AI). An initial state is prepared to be a localized wave function $|\psi_0\rangle \propto \sum_{x=1}^{L} e^{-x}\,|x\rangle$, and the evolutions of the population (normalized intensity) $|\langle x|\psi_t\rangle|^2 / \langle\psi_t|\psi_t\rangle$ are shown for (a) $|x\rangle = |a_1\rangle$, (b) $|x\rangle = |b_1\rangle$, (c) $|x\rangle = |a_2\rangle$ and (d) $|x\rangle = |b_2\rangle$ [see Fig. 3 (a) in the main text for details]. The insulator with $L = 50$ sites is characterized by the parameters $t = \delta = 1.0$ and $\gamma = 0.2$ for the topological phase (blue curves) and $\gamma = 3.0$ for the trivial phase (orange curves). In the topological phase, there emerges time-reversal-symmetry-protected topological edge states whose imaginary parts vanish. As a consequence, the population of the wave function in the topological phase is greater than that in the trivial phase.

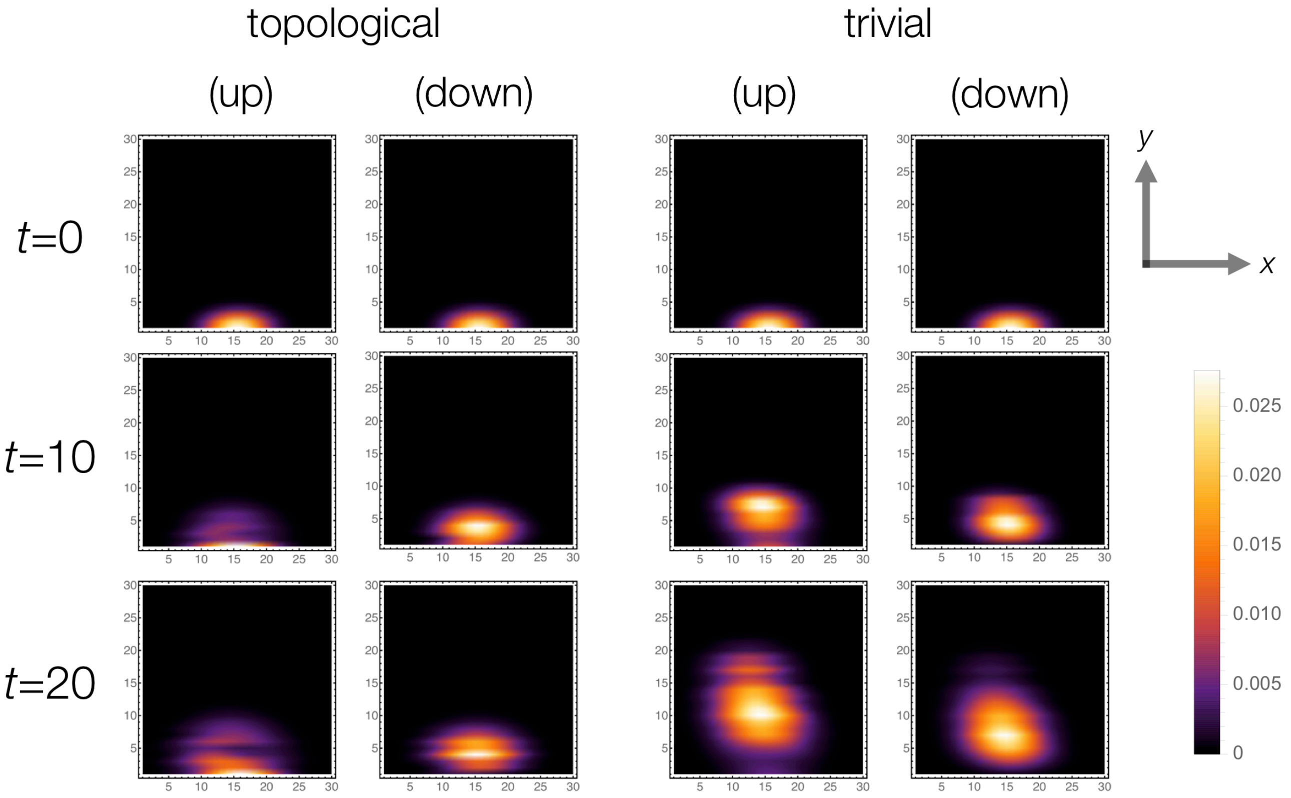

Supplementary Figure 10. Population dynamics in the non-Hermitian quantum spin Hall insulator (2D class AII). The insulator with 30 $\times$ 30 sites is characterized by the parameters $t = 1.0$, $m = -1.0$, $\lambda = 0.5$, $\gamma = 0.8$ for the topological phase (left) and $t = 1.0$, $m = 3.0$, $\lambda = 0.8$, $\gamma = 1.2$ for the trivial phase (right). An initial state is a localized wave function $|\psi_0\rangle \propto \sum_{x,y} e^{-(x-15.5)^2/36-(y-1)^2/9} |x, y\rangle$, and the evolutions of the population (normalized intensity) $|\langle x, y|\psi_t\rangle|^2 / \langle \psi_t|\psi_t\rangle$ are shown at $t = 0, 10, 20$. In the topological phase, the wave packet remains localized due to the presence of the helical edge states. In contrast to the Hermitian quantum spin Hall insulator, the spin-up component is more localized than the spin-down component due to the presence of the imaginary gap between the helical edge states. Moreover, the wave packet does not move along the edges since the real parts of the energy dispersions are flat for the helical edge states. In the trivial phase, on the other hand, the wave packet quickly diffuses into the bulk due to the absence of the edge states.

the dynamics of non-Hermitian topological systems. When an initial state is prepared to be

$$|\psi_0\rangle = \sum_n c_n |\varphi_n\rangle, \quad c_n = \langle \chi_n|\psi_0\rangle, \tag{24}$$

where $|\varphi_n\rangle$ ($|\chi_n\rangle$) is a right (left) eigenstate of the non-Hermitian Hamiltonian [20], the wave function evolves as

$$|\psi_t\rangle = e^{-\mathrm{i}Ht} |\psi_0\rangle = \sum_n c_n e^{-\mathrm{i}E_n t} |\varphi_n\rangle. \tag{25}$$

Although the system eventually reaches a stationary state $|\varphi_n\rangle$ with the largest imaginary part of E_n, unique topological features appear in the non-Hermitian transient dynamics, which have been observed in recent experiments [15–18].

For the non-Hermitian topological insulator given by Eq. (3) in the main text, the imaginary parts of the eigenenergies of the topologically protected edge states vanish due to the presence of time-reversal symmetry. Consequently, these edge states are stable, despite instability of the bulk states. The presence of such stable edge states can be detected by focusing on the evolution of the particle population near the edges. In fact, when an initial state is prepared as a localized state at one edge, its population near the edge in the topological phase is greater than that in the trivial phase (Supplementary Figure 9).

For the non-Hermitian quantum spin Hall insulator given by Eq. (7) and shown in Fig. 5 in the main text, the helical edge states emerge corresponding to the non-trivial topology of the bulk. As a consequence, when an initial state is prepared as a localized state at one edge, the wave packet remains localized near the edge in the topological phase (Supplementary Figure 10). In the trivial phase, on the other hand, the wave packet quickly diffuses into the bulk due to the absence of the edge states. There are several significant distinctions between the Hermitian and non-Hermitian quantum spin Hall insulators. In contrast to the

Hermitian one, the spin-up component is more localized than the spin-down component due to the presence of the imaginary gap between the helical edge states [see Fig. 4 (b) in the main text]. Moreover, the wave packet does not move along the edges since the real parts of the energy dispersions are flat for the helical edge states [see Fig. 4 (a) in the main text].

SUPPLEMENTARY NOTE 9: BULK-EDGE CORRESPONDENCE IN NON-HERMITIAN SYSTEMS

The bulk-edge correspondence in Hermitian topological systems is generally understood with a continuum model described by a Dirac Hamiltonian [21, 22]. Although the topological classification is drastically altered by non-Hermiticity as demonstrated in the main text, the bulk-edge correspondence can be generalized to a non-Hermitian continuum model in the same manner as the Hermitian one. We here consider a one-dimensional Dirac Hamiltonian that respects chiral symmetry $\mathcal{S} := \sigma_z$ (hence belongs to 1D class AIII):

$$H(k) = (k + \mathrm{i}g)\,\sigma_x + (m + \mathrm{i}\delta)\,\sigma_y, \tag{26}$$

where k is a wavenumber (momentum), and $g,\, m,\, \delta \in \mathbb{R}$ are real parameters that characterize the topological or trivial phase. The energy dispersion of this continuum model is obtained as

$$E(k) = \pm\sqrt{(k + \mathrm{i}g)^2 + (m + \mathrm{i}\delta)^2} = \pm\sqrt{k^2 + m^2 - g^2 - \delta^2 + 2\mathrm{i}\,(kg + m\delta)}, \tag{27}$$

and hence the complex bands are gapped for $|m| > |g|$. As with the Hermitian case, the topological invariant (winding number) can be defined for the gapped phases as

$$W = \int_{-\infty}^{\infty} \frac{dk}{4\pi\mathrm{i}}\,\mathrm{tr}\left[\mathcal{S}H^{-1}\frac{dH}{dk}\right] = -\int_{-\infty}^{\infty} \frac{dk}{2\pi}\frac{m + \mathrm{i}\delta}{(k + \mathrm{i}g)^2 + (m + \mathrm{i}\delta)^2} = -\frac{\mathrm{sgn}\,[m]}{2}. \tag{28}$$

Here the half-integer topological invariant W is common to the continuum models and should be complemented by the structure of wave functions away from the Dirac point [23, 24].

To investigate the emergence of topologically protected boundary states, we consider a domain wall defined by

$$g(x) = g_+\theta(x) + g_-\theta(-x)\,, \quad m(x) = m_+\theta(x) + m_-\theta(-x)\,, \quad \delta(x) = \delta_+\theta(x) + \delta_-\theta(-x)\,, \tag{29}$$

with a step function $\theta(x)$. Then we solve the Schrödinger equation

$$\left[\left(-\mathrm{i}\frac{d}{dx} + \mathrm{i}g(x)\right)\sigma_x + (m(x) + \mathrm{i}\delta(x))\,\sigma_y\right]\varphi(x) = 0 \tag{30}$$

to find the topologically protected boundary states with zero energy. For $x > 0$, if we take an ansatz $\varphi_+(x) = (a\ b)^T\, e^{-x/\xi_+}$, the above Schrödinger equation reduces to

$$\left(\xi_+^{-1} + g_+ + m_+ + \mathrm{i}\delta_+\right) a = \left(\xi_+^{-1} + g_+ - m_+ - \mathrm{i}\delta_+\right) b = 0. \tag{31}$$

We notice that $\mathrm{Re}\,[\xi_+] > 0$ is needed for the localization of the boundary states. Then, for $m_+ > 0$, we have $g_+ + m_+ > 0$ and hence $\mathrm{Re}\left[\xi_+^{-1} + g_+ + m_+ + \mathrm{i}\delta_+\right] > 0$; for $m_+ < 0$, we have $g_+ - m_+ > 0$ and hence $\mathrm{Re}\left[\xi_+^{-1} + g_+ - m_+ - \mathrm{i}\delta_+\right] > 0$. Therefore, we obtain

$$\begin{aligned} \varphi_+(x) &= \begin{pmatrix} 0 \\ 1 \end{pmatrix} \exp\left[-\left(m_+ - g_+ + \mathrm{i}\delta_+\right)x\right] \quad \text{for} \quad m_+ > 0; \\ \varphi_+(x) &= \begin{pmatrix} 1 \\ 0 \end{pmatrix} \exp\left[-\left(-m_+ - g_+ - \mathrm{i}\delta_+\right)x\right] \quad \text{for} \quad m_+ < 0. \end{aligned} \tag{32}$$

For $x < 0$, on the other hand, we obtain

$$\begin{aligned} \varphi_-(x) &= \begin{pmatrix} 1 \\ 0 \end{pmatrix} \exp\left[-\left(m_- + g_- + \mathrm{i}\delta_-\right)x\right] \quad \text{for} \quad m_- > 0; \\ \varphi_-(x) &= \begin{pmatrix} 0 \\ 1 \end{pmatrix} \exp\left[-\left(-m_- + g_- - \mathrm{i}\delta_-\right)x\right] \quad \text{for} \quad m_- < 0. \end{aligned} \tag{33}$$

Due to the boundary condition $\varphi_+(0) = \varphi_-(0)$, the bound states emerge if and only if the signs of m_+ and m_- are different, reflecting the different winding numbers in each region; the emergence of the topologically protected bound states corresponds to the non-trivial topology of the bulk. Notably, similar discussions for non-Hermitian continuum models in two dimensions are found in Supplementary References [25, 26].

[1] Misra, B. & Sudarshan, E. C. G. The Zeno's paradox in quantum theory. *J. Math. Phys.* **18**, 756-763 (1977).
[2] Facchi, P. & Pascazio, S. Quantum Zeno subspaces. *Phys. Rev. Lett.* **89**, 080401 (2002).
[3] Kwiat, P., Weinfurter, H., Herzog, T., Zeilinger, A. & Kasevich, M. A. Interaction-free measurement. *Phys. Rev. Lett.* **74**, 4763-4766 (1995).
[4] Syassen, M., Bauer, D. M., Lettner, M., Volz, T., Dietze, D., García-Ripoll, J. J., Cirac, J. I., Rempe, G. & Dürr, S. Strong dissipation inhibits losses and induces correlations in cold molecular gases. *Science* **320**, 1329-1331 (2008).
[5] Barontini, G., Labouvie, R., Stubenrauch, F., Vogler, A., Guarrera, V. & Ott, H. Controlling the dynamics of an open many-body quantum system with localized dissipation. *Phys. Rev. Lett.* **110**, 035302 (2013).
[6] Gong, Z., Higashikawa, S. & Ueda, M. Zeno Hall effect. *Phys. Rev. Lett.* **118**, 200401 (2017).
[7] Bender, C. M. & Boettcher, S. Real spectra in non-Hermitian Hamiltonians having $\mathcal{PT}$ symmetry. *Phys. Rev. Lett.* **80**, 5243-5246 (1998).
[8] Malzard, S., Poli, C., & Schomerus, H. Topologically protected defect states in open photonic systems with non-Hermitian charge-conjugation and parity-time symmetry. *Phys. Rev. Lett.* **115**, 200402 (2015).
[9] Ge, L. Symmetry-protected zero-mode laser with a tunable spatial profile. *Phys. Rev. A* **95**, 023812 (2017).
[10] Kawabata, K., Ashida, Y., Katsura, H. & Ueda, M. Parity-time-symmetric topological superconductor. *Phys. Rev. B* **98**, 085116 (2018).
[11] Kitaev, A. Y. Unpaired Majorana fermions in quantum wires. *Phys.-Usp.* **44**, 131-136 (2001).
[12] Peng, B., Özdemir, Ş. K., Liertzer, M., Chen, W., Kramer, J., Yılmaz, H., Wiersig, J., Rotter, S. & Yang, L. Chiral modes and directional lasing at exceptional points. *Proc. Natl. Acad. Sci. USA* **113**, 6845-6850 (2016).
[13] Chen, W., Özdemir, Ş. K., Zhao, G., Wiersig, J. & Yang, L. Exceptional points enhance sensing in an optical microcavity. *Nature* **548**, 192-196 (2017).
[14] Fu, L. & Kane, C. L. Topological insulators with inversion symmetry. *Phys. Rev. B* **76**, 045302 (2007).
[15] Xiao, L., Zhan, X., Bian, Z. H., Wang, K. K., Zhang, X., Wang, X. P., Li, J., Mochizuki, K., Kim, D., Kawakami, N., Yi, W., Obuse, H., Sanders, B. C. & Xue, P. Observation of topological edge states in parity-time-symmetric quantum walks. *Nat. Phys.* **13**, 1117-1123 (2017).
[16] St-Jean, P., Goblot, V., Galopin, E., Lemaître, A., Ozawa, T., Le Gratiet, L., Sagnes, I., Bloch, J. & Amo, A. Lasing in topological edge states of a one-dimensional lattice. *Nat. Photon.* **11**, 651-656 (2017).
[17] Parto, M., Wittek, S., Hodaei, H., Harari, G., Bandres, M. A., Ren, J., Rechtsman, M. C., Segev, M., Christodoulides, D. N. & Khajavikhan, M. Edge-mode lasing in 1D topological active arrays. *Phys. Rev. Lett.* **120**, 113901 (2018).
[18] Harari, G., Bandres, M. A., Lumer, Y., Rechtsman, M. C., Chong, Y. D., Khajavikhan, M., Christodoulides, D. N. & Segev, M. Topological insulator laser: theory. *Science* **359**, eaar4003 (2018); Bandres, M. A., Wittek, S., Harari, G., Parto, M., Ren, J., Segev, M., Christodoulides, D. & Khajavikhan, M. Topological insulator laser: experiments. *Science* **359**, eaar4005 (2018).
[19] Lee, T. E. Anomalous edge state in a non-Hermitian lattice. *Phys. Rev. Lett.* **116**, 133903 (2016).
[20] Brody, D. C. Biorthogonal quantum mechanics. *J. Phys. A* **49**, 10LT03 (2016).
[21] Bernevig, B. A. & Hughes, T. L. *Topological insulators and topological superconductors* (Princeton University Press, 2013).
[22] Asbóth, J. K., Oroszlány, L. & Pályi, A. *A short course on topological insulators*. *Lecture Notes in Physics* **919** (Springer, 2016).
[23] Haldane, F. D. M. Model for a quantum Hall effect without Landau levels: Condensed-matter realization of the "parity anomaly." *Phys. Rev. Lett.* **61**, 2015 (1988).
[24] Schnyder, A. P., Ryu, S., Furusaki, A. & Ludwig, A. W. W. Classification of topological insulators and superconductors in three spatial dimensions. *Phys. Rev. B* **78**, 195125 (2008).
[25] Leykam, D., Bliokh, K. Y., Huang, C., Chong, Y. D. & Nori, F. Edge modes, degeneracies, and topological numbers in non-Hermitian systems. *Phys. Rev. Lett.* **118**, 040401 (2017).
[26] Shen, H., Zhen, B. & Fu, L. Topological band theory for non-Hermitian Hamiltonians. *Phys. Rev. Lett.* **120**, 146402 (2018).
