# Supplementary material for: Topological unification of time-reversal and particle-hole symmetries in non-Hermitian physics
Source: Nat Commun. 2019 Jan 17;10:297. doi: 10.1038/s41467-018-08254-y (PMC6336874; doi:10.1038/s41467-018-08254-y)
Supplement: Supplementary file 2 — Peer Review File [file 41467_2018_8254_MOESM2_ESM.docx]

**Editorial Note:** This manuscript has been previously reviewed at another journal that is not operating a transparent peer review scheme. The manuscript was considered suitable for publication without further review at Nature Communications.
